# ECPD-MIE: An adaptive entropy-controlled chaotic permutation-diffusion scheme for secure multi-image encryption of grayscale and color images

**DOI:** 10.1371/journal.pone.0357571

**Published:** 2026-09-11

**Authors:** Bibhuti Bhusan Mishra, K. Abhimanyu Kumar Patro, Sudhakar Das

**Affiliations:** 1 Department of Electronics and Communication Engineering, NIST University, Berhampur, Odisha, India; 2 Manipal Institute of Technology, Manipal Academy of Higher Education, Manipal, India; Deakin University, AUSTRALIA

## Abstract

The rapid growth of multimedia communication has significantly increased the demand for robust encryption techniques capable of securely handling heterogeneous image data. Conventional image encryption schemes typically focus on either grayscale or color images independently, thereby limiting their applicability in real-world scenarios that require simultaneous protection of mixed image types. To address this limitation, this paper proposes ECPD-MIE, an adaptive entropy-controlled chaotic permutation-diffusion scheme for secure multi-image encryption of grayscale and color images within a unified framework. In the proposed method, multiple grayscale and color images of arbitrary dimensions are first adaptively normalized and concatenated into a composite structure, enabling simultaneous encryption while preserving their structural characteristics. High-quality pseudo-random sequences generated from multiple parallel piecewise linear chaotic maps (PWLCMs) are employed to perform circular row and column permutations, ensuring strong confusion properties. To enhance plaintext sensitivity and key security, dynamic initial conditions are derived using SHA-256 hashing of the composite image. Furthermore, an entropy-controlled cross-bit-plane diffusion mechanism is introduced to adaptively improve randomness and diffusion across grayscale intensities and color channels. Comprehensive security analyses demonstrate uniform histogram distributions, near-zero correlations among adjacent pixels, and information entropy values close to the theoretical maximum. The proposed method achieves an average NPCR of 99.62%, UACI of 33.47%, and entropy approaching 8, indicating strong resistance against differential and statistical attacks. Additionally, the encrypted images successfully pass the NIST SP800−22 randomness tests. The scheme exhibits low computational complexity of O(M×N) and supports heterogeneous image datasets, making it a secure and efficient solution for modern multimedia communication systems.

## Introduction

The swift evolution of digital communication technology along with easy access to multimedia technology has led to the explosive growth of production, distribution, and storage of visual content. Visual images play a crucial part in many areas such as telemedicine, cloud computing, remote sensing, and social media. The open and distributed nature of modern communication systems makes multimedia information vulnerable to various types of security attacks, including unauthorized access, interception, modification, and leakage. Hence, the need for information security has become a primary issue with regards to image information.

Conventional cryptographic algorithms, such as Advanced Encryption Standard (AES), Data Encryption Standard (DES), Rivest-Shamir-Adleman (RSA), etc., are primarily designed to work with textual or binary data [1,2]. Though these algorithms provide good security, they are often found to be less efficient when used to encrypt images directly because of the inherent nature of images, which are characterized by large amounts of data, a high degree of redundancy, and strong correlation between neighboring pixels [3–5]. All of this has resulted in the need to design image encryption algorithms that are effective in destroying the correlation between pixels while being efficient in computation.

Among the varied methodologies, chaos-based encryption techniques have attracted much interest due to their inherent properties, which include high sensitivity to initial conditions, ergodicity, pseudo-randomness, and unpredictability [6–9]. These properties are quite similar to the basic demands of any cryptographic technique. In a conventional chaos-based image encryption technique, a permutation-diffusion scheme is commonly used, in which pixel positions are permuted to reduce spatial correlation, and pixel values are diffused to spread the confusion throughout the whole image.

To cope with the fast emergence of multimedia data, multi-image encryption technology has come into existence as an efficient means of encrypting several images at a time. Not only is this approach efficient, but it can improve the level of security of the images. Most of the image encryption schemes that have been devised till now work on either grayscale or color images. Multimedia data used in real-time often consists of a combination of grayscale and color images of different sizes.

Various sophisticated mechanisms have been used to strengthen the security of image encryption algorithms, including plaintext-dependent key generation, adaptive diffusion, DNA-inspired operations, and lightweight chaos-based encryption techniques. Hash functions enable the generation of dynamic plaintext-dependent parameters, thereby improving resistance against known-plaintext and chosen-plaintext attacks. Similarly, adaptive diffusion mechanisms improve the propagation of pixel modifications throughout the ciphertext. Although chaotic permutation-diffusion structures have been extensively investigated in the literature, recent research has focused on enhancing their effectiveness through adaptive control strategies, DNA-based operations, and lightweight encryption architectures. These developments highlight the continuing need for image encryption frameworks that simultaneously achieve high security, computational efficiency, and flexibility for heterogeneous image sets. Hash functions allow for the creation of dynamic keys, which depends on the plaintext, and makes it harder for the attacker to conduct known plaintext and chosen plaintext attacks. Adaptive diffusion helps to improve pixel change spreading.

Furthermore, the idea of secure multimedia communication is very important in accomplishing the following goals and ambitions related to technology and society, especially the improvement of sustainable digital infrastructure and digital systems as per United Nations’ Sustainable Development Goal 9 (SDG 9) and Sustainable Development Goal 16 (SDG 16). Therefore, efficient image encryption systems are fundamental in building a trusted digital ecosystem.

Inspired by the above challenges, this paper proposes a novel framework called ECPD-MIE, which is an adaptive entropy-controlled chaotic permutation-diffusion method for secure multi-image encryption of grayscale and color images. The proposed method adopts a unified framework for image processing, in which multiple grayscale and color images of arbitrary dimensions are normalized and integrated into a composite structure for simultaneous encryption. Pseudo-random sequences of high quality are employed for circular row and column permutations of images, which effectively reduce pixel correlations in images. To improve plaintext and key security, dynamic initial values are generated by the SHA-256 hash function. Meanwhile, an entropy-controlled cross-bit-plane diffusion method is proposed for adaptive improvement of randomness and diffusion properties in grayscale and color images.

Contrary to multi-image encryption algorithms designed specifically for homogenous images, the algorithm developed in this research is a more general solution for heterogeneous images due to its ability to encrypt multiple images in one go. The addition of entropy-based diffusion and hashing further improves the security.

The contributions made by this research can be listed as follows:

- An integrative and adaptive entropy-controlled chaotic permutation-diffusion technique is proposed for secure multi-image encryption, allowing simultaneous encryption of grayscale and color images of arbitrary sizes.
- Multiple parallel PWLCM systems are utilized to produce high-quality pseudo-random sequences for circular row and column permutations, improving the confusion effect and reducing pixel correlation.
- A dynamic key generation technique using the SHA-256 algorithm is proposed to produce plaintext-dependent encryption keys, greatly improving the sensitivity of the encryption keys and the security against cryptanalytic attacks.
- An entropy-controlled adaptive diffusion mechanism is integrated with plaintext-dependent PWLCM-generated chaotic masks to enhance diffusion performance within a unified heterogeneous multi-image encryption framework.
- Experimental results show that the proposed encryption technique has robust encryption performance with average NPCR of 99.62%, UACI of 33.47%, and entropy values approaching the theoretical limits, as well as uniform histograms, zero-pixel correlation, and NIST SP800−22 test suite results.

The remainder of this paper is organized as follows. First, the related work on existing image encryption techniques is reviewed. Next, the fundamental concepts and preliminaries required for understanding the proposed method are presented. The proposed multi-image encryption methodology is then described in detail. Subsequently, the simulation results and comprehensive security analyses are discussed to evaluate the performance of the proposed scheme. Finally, the paper concludes with a summary of the findings and directions for future research.

## Related work

With the development of multimedia communication technology and internet services, the secure transmission of digital images is an increasingly important problem. However, existing encryption algorithms, which are mainly used to encrypt textual data, are not very effective in image encryption, considering the nature of image data, such as the large data size, high redundancy, and strong correlation between neighboring pixels. To overcome these difficulties, a variety of image encryption algorithms have been proposed, among which chaos-based image encryption algorithms have drawn particular attention owing to their sensitivity to initial values, ergodicity, and pseudo-randomness.

In the initial phase, the focus was on the encryption of a single image, where chaotic maps were used for performing the process of permutation and diffusion, leading to the disruption of the relationship between pixels, thus improving the effectiveness of the encryption [10–14]. While the results of the encryption process were impressive, these results were not very effective when it came to the encryption of several images due to the increased costs associated with encrypting each image separately.

In the beginning, MIE algorithms were combined with chaotic permutation-diffusion algorithms through bit plane slicing to ensure better security of the encryption algorithms [15]. Later on, researchers developed chaotic map-based MIE schemes in order to improve the randomness and computational complexity of the algorithmic process [16,17]. However, such algorithms were seen to be characterized by restricted diffusion performance as well as a small key space.

To overcome such shortcomings, researchers have devised several multi-level permutation schemes designed to improve spatial diffusion as well as decrease pixel correlation. For instance, secure multi-level permutation schemes have been invented that improve randomness as well as the efficiency of encryption [18]. At the same time, DNA-based image encryption schemes have been combined with chaos theory, where pixels are converted into DNA codes, which are then processed using biological techniques to increase confusion and diffusion [19]. Although they provide enhanced security, these algorithms are often computationally expensive.

One of the notable trends in MIE research is the application of cross-coupled chaotic systems along with image fusion methods. For example, Patro et al. [20] presented a dual-layer cross-coupled chaotic mapping architecture for performing synchronized permutation and diffusion processes, leading to increased security and performance. Further research has utilized hash functions in generating plaintext-dependent keys by employing chaos-based key generation techniques [21].

More recently, researchers have explored the integration of advanced chaotic systems, adaptive control strategies, and DNA-inspired operations to further enhance image encryption performance. Roohi et al. [22] developed a cryptographic framework based on adaptive model-free synchronization of fractional-order neural networks and demonstrated its applicability to secure color image encryption and transmission. In addition, DNA-inspired image encryption techniques have received considerable attention owing to their ability to provide enhanced confusion and diffusion characteristics. For example, Mirzajani et al. [23] proposed a color image encryption algorithm utilizing DNA subsequence operations together with chaotic permutation and adaptive diffusion mechanisms. Furthermore, lightweight chaos-based image encryption schemes have been developed for resource-constrained applications. Chen et al. [24] introduced a selective chaos-driven encryption technique for medical image protection that combines adaptive region selection with permutation-diffusion operations to improve both security and computational efficiency [22–24].

In order to increase the complexity involved in encryption, studies have been undertaken in 3D image encryption, as well as multi-dimensional image encryption techniques. This technique uses several images in creating higher dimensional data structures, which involve carrying out the process of spatial scrambling using chaos sequences [25,26]. The technique is able to provide greater security against attacks through statistical analysis, although at the cost of increased computation.

Recently, a significant effort has been made to improve the efficiency and security of different multimedia image encryption schemes. A fast permutation and diffusion approach has been adopted to increase the efficiency of MIE schemes, without hampering the effectiveness of the same [27,28]. The implementation of cross plane encryption schemes is another way of increasing the efficiency of image encryption schemes, through the diffusion of pixels across several planes before the process of encryption [29]. Quaternion transforms and other transform-domain approaches have been used to improve the efficiency of image encryption schemes, making it possible to encrypt color images’ channels under one mathematical model [30]. In addition, there are specific encryption schemes used for special cases, like the encryption of medical images [31].

In order to overcome shortcomings of traditional chaotic systems, modern chaotic maps have been developed which possess higher levels of randomness along with larger key spaces. The exponent sine cosine chaotic systems have been used in the development of such chaotic maps [32]. Color image encryption algorithms using these chaotic maps have been designed to enhance security in color images [33]. Parallel encryption has also been suggested in order to achieve encryption of multiple images simultaneously [34]. Spatiotemporal chaotic systems have been suggested for improved security of color images [35, 36].

In recent researches conducted in this area, there have been attempts at incorporating high dimensional chaos in the design of MIE schemes in an attempt to increase their efficiency and security in the encryption process. For instance, Zhou et al. [37] developed a four-dimensional chaos scheme along with multi-layer embedding resulting in improved encryption performance. There have also been designs of fast, parallel chaotic encryption schemes which have relied on hyper-chaos as well as memristors in an attempt to satisfy real-time encryption requirements [38]. Besides, new designs have incorporated machine learning as well as data compression in an effort to improve the efficiency of the encryption algorithm. This is evident in the design of chaotic encryption that relies on neural networks in compressing image data [39]. High speed encryption schemes have been achieved through hardware implementations, using FPGA technology [40].

Despite these developments, including recent advances in chaos synchronization-based cryptography, DNA-inspired image encryption, and lightweight selective image protection schemes, several challenges remain in current multi-image encryption systems. Many existing methods rely on computationally intensive operations such as DNA encoding, compressive sensing, transform-domain processing, or high-dimensional chaotic systems, which increase implementation complexity. Furthermore, most existing schemes are designed for either grayscale images or color images individually and do not provide a unified framework capable of efficiently processing heterogeneous image sets of arbitrary dimensions. Although permutation-diffusion architectures have been widely studied, achieving an effective balance among security, diffusion capability, computational efficiency, plaintext sensitivity, and heterogeneous multi-image processing remains an open research challenge.

Motivated by these limitations, this paper presents a novel adaptive entropy-controlled chaotic permutation-diffusion framework, referred to as ECPD-MIE, for the secure encryption of heterogeneous image sets. While chaotic permutation and adaptive diffusion have been investigated individually in previous studies, the proposed contribution lies in the integration of adaptive image concatenation, SHA-256-based plaintext-dependent chaotic perturbation, independent PWLCM-based row and column permutation, and entropy-controlled adaptive diffusion within a unified framework capable of simultaneously processing grayscale and color images of arbitrary dimensions. Furthermore, the proposed composite-image architecture enables single-pass multi-image encryption, thereby improving scalability while maintaining strong security characteristics.

### Preliminaries

This section describes the fundamental ideas and mathematical tools used in the suggested encryption system, which include the piecewise linear chaotic map, the SHA-256 cryptographic hash function, the permutation and diffusion method, and Shannon entropy as a randomness indicator.

### Piecewise Linear Chaotic Map (PWLCM)

The use of chaos in image encryption can be attributed to the features that come with the systems such as sensitivity to changes in the initial conditions, ergodicity, pseudo-randomness, and determinism. Piecewise Linear Chaotic Map (PWLCM) has received much attention in recent times owing to the fact that it is mathematically simple and easy to compute.

The PWLCM is described by Eq (1) as:


$$\begin{array}{c} x_{n+1}=\left\{ \begin{array}{cc} \frac{x_{n}}{p}, & 0\leq x_{n}<p\\ \frac{x_{n}-p}{0.5-p}, & p\leq x_{n}<0.5\\ \frac{1-p-x_{n}}{0.5-p}, & 0.5\leq x_{n}<1-p\\ \frac{1-x_{n}}{p}, & 1-p\leq x_{n}<1 \end{array}\right. \end{array}$$ (1)

where $x_{n}\in \left(0,1\right)$ represents the state variable at the *n*-th iteration, and p∈(0,0.5) is the control parameter. When *p* lies within this interval, the system exhibits strong chaotic behavior and generates sequences with excellent pseudo-random properties.

Due to these advantages, it is widely used for generating pseudo-random sequences for image encryption by applying the PWLCM. In our proposed method, multiple PWLCM systems with different initial values and control parameters are used for generating independent chaotic sequences for permutation and diffusion operations [41,42].

### SHA-256 cryptographic hash function

Cryptographic hash functions play a vital role in enhancing the security of encryption methods by producing dynamic keys, which depend on the plaintext. The Secure Hash Algorithm 256 (SHA-256) produces a 256-bit fixed-size hash value, which depends on the input of any arbitrary size.

The process of hashing can be represented as:


$$\begin{array}{c} H=SHA256\left(M\right) \end{array}$$

where *M* represents the input message (or image data), and *H* denotes the resulting 256-bit hash value.

SHA-256 possesses some important cryptographic properties, such as the avalanche effect, collision resistance, and preimage resistance. These properties guarantee a considerable change in the output hash values even with a small change in the input.

In the proposed scheme, the hash value obtained by using the SHA-256 algorithm on the combined image is used to derive the initial conditions and control parameters of the chaotic systems. This has created a dependency on the plaintext, thus making the scheme more robust against known and chosen plaintext attacks.

## Permutation-diffusion architecture

Most chaos-based image encryption methods use a permutation-diffusion architecture to achieve the confusion and diffusion of the plaintext image. The permutation part rearranges the spatial positions of the pixels in the image, and the diffusion part changes the pixel values in a way that the noise spreads throughout the entire image.

The plaintext image is assumed to be represented as:


$$\begin{array}{c} P=\left\{p(i,j)\right\},\;1\leq i\leq M,\;1\leq j\leq N \end{array}$$

where p(i,j) denotes the pixel value at position (i,j), and M×N represents the image size.

After permutation, the shuffled image is expressed as:


$$\begin{array}{c} P^{\prime }=Permute\left(P\right) \end{array}$$

The diffusion process produces the final encrypted image as:


$$\begin{array}{c} C\left(i,j\right)=P^{\prime }\left(i,j\right)⊕D\left(i,j\right) \end{array}$$

where D(i,j) represents the diffusion sequence generated by chaotic systems, and ⊕ denotes the bitwise XOR operation.

This combination of permutation and diffusion is a robust protection against statistical and differential attacks. In the proposed ECPD-MIE scheme, the circular permutation of rows and columns is carried out using PWLCM-based sequences, followed by the proposed entropy-controlled cross-bit-plane diffusion mechanism.

### Shannon entropy

Shannon entropy is usually used to measure the randomness of encrypted images. A good encryption algorithm should produce encrypted images whose Shannon entropy is close to the maximum possible.

Shannon entropy is defined by Eq (2) as:


$$\begin{array}{c} H(m)=-\sum_{i=0}^{255}P(m_{i})\log_{2}P\left(m_{i}\right) \end{array}$$ (2)

where $P\left(m_{i}\right)$ denotes the probability of occurrence of the pixel intensity $m_{i}$.

For an ideal 8-bit image, the maximum entropy is:


$$\begin{array}{c} H_{max}=8 \end{array}$$

An entropy value close to 8 indicates a uniform distribution of pixel intensities, which reflects a high level of randomness in the encrypted image. Therefore, Shannon entropy is an essential tool in measuring the efficacy of image encryption algorithms.

In this research, entropy analysis is used to test the level of randomness in the encrypted image to validate the efficacy of the proposed entropy-controlled diffusion mechanism.

### Proposed methodology

The proposed encryption scheme, referred to as ECPD-MIE, is designed for the simultaneous encryption of multiple heterogeneous images, including both grayscale and color images. The framework consists of three major phases: key generation, encryption, and decryption. During the key generation phase, the input images are combined to form a composite image, and a SHA-256 digest is generated. Rather than serving as an independent secret key, the SHA-256 digest acts as a plaintext-dependent perturbation mechanism that dynamically modifies the initial conditions and control parameters of multiple PWLCM systems, thereby enhancing plaintext sensitivity and generating highly random chaotic sequences.

During the encryption phase, grayscale and color images are first processed separately and subsequently combined into composite images. Chaotic row and column permutation operations are then performed using two independent PWLCM-generated sequences. The row and column permutations collectively provide a two-dimensional scrambling effect that significantly reduces the spatial correlation among adjacent pixels and improves the confusion property of the cryptosystem. Furthermore, the SHA-256-based perturbation mechanism dynamically modifies the chaotic parameters for different plaintext images, resulting in distinct permutation patterns for different image sets. After permutation, an entropy-controlled diffusion process is carried out using additional PWLCM-generated chaotic masks. In this stage, adaptive chaotic masks are combined with pixel values through bitwise XOR operations, while the entropy-based weight factor serves as a plaintext-dependent adaptive diffusion parameter. Finally, the encrypted composite image is separated to obtain the corresponding individual cipher images.

The decryption phase is the exact inverse of the encryption procedure. Initially, the received cipher images are recombined to reconstruct the encrypted composite image. Using the same secret chaotic parameters together with the SHA-256-based perturbation mechanism, the chaotic permutation sequences and adaptive diffusion masks are regenerated. The encrypted composite image is then subjected to inverse diffusion and inverse permutation operations to recover the original image arrangement. Finally, the reconstructed composite image is separated to obtain the original grayscale and color images without any loss of information.

A detailed explanation of the individual stages involved in the proposed ECPD-MIE framework is provided below.

### Key generation

In the proposed ECPD-MIE system, the secret key parameter values are generated based on a dynamic method dependent on the plaintext. This significantly improves the sensitivity of the key value while ensuring that the keys generated are resilient against cryptanalysis attacks.

1. Suppose there are *I* numbers of input images to encrypt. The first stage involves combining all the input images to generate an image composite referred to as *IGC*.
2. The SHA-256 cryptographic hash function is then used on the composite image to obtain a hash value in bits of 256 represented by the hexadecimal sequence:

$$\begin{array}{c} HX=\left(HX_{1},HX_{2},\cdots ,HX_{64}\right) \end{array}$$

where $HX_{i}\left(i=1,2,...,64\right)$ is the $i^{th}$ hexadecimal value of the hash sequence.

3. These hash values are subsequently used to update the initial parameters of five independent PWLCM systems. This process introduces strong randomness and ensures that even a slight variation in the input images produces completely different chaotic sequences.

It is important to note that the SHA-256 digest is not employed as an independent secret key. Instead, it acts as a plaintext-dependent perturbation mechanism that dynamically modifies the initial conditions and control parameters of the PWLCM systems. Consequently, even a slight modification in the composite image produces a substantially different hash digest, resulting in different chaotic sequences for permutation and diffusion. This property enhances plaintext sensitivity and improves resistance against known-plaintext and chosen-plaintext attacks.

The updated parameters of PWLCM-1 are calculated by Eq (3) as:


$$\begin{array}{c} \left\{ \begin{array}{c} v\left(1\right)=v_{1}-\left(\begin{array}{c} \left(\frac{{HX}_{1}+{HX}_{2}+\cdots +{HX}_{6}}{10^{15}}\right)-\\ \left\lceil \frac{{HX}_{1}+{HX}_{2}+\cdots +{HX}_{6}}{10^{15}}\right\rceil \end{array}\right)\times 0.01\\ aa=a_{1}-\left(\begin{array}{c} \left(\frac{{HX}_{7}+{HX}_{8}+\cdots +{HX}_{12}}{10^{15}}\right)-\\ \left\lceil \frac{{HX}_{7}+{HX}_{8}+\cdots +{HX}_{12}}{10^{15}}\right\rceil \end{array}\right)\times 0.01 \end{array}\right. \end{array}$$ (3)

Similarly, the updated parameters of PWLCM-2 are obtained by Eq (4) as:


$$\begin{array}{c} \left\{ \begin{array}{c} w\left(1\right)=w_{1}-\left(\begin{array}{c} \left(\frac{{HX}_{13}+{HX}_{14}+\cdots +{HX}_{18}}{10^{15}}\right)-\\ \left\lceil \frac{{HX}_{13}+{HX}_{14}+\cdots +{HX}_{18}}{10^{15}}\right\rceil \end{array}\;\right)\times 0.01\\ bb=b_{1}-\left(\begin{array}{c} \left(\frac{{HX}_{19}+{HX}_{20}+\cdots +{HX}_{24}}{10^{15}}\right)-\\ \left\lceil \frac{{HX}_{19}+{HX}_{20}+\cdots +{HX}_{24}}{10^{15}}\right\rceil \end{array}\;\right)\times 0.01 \end{array}\right. \end{array}$$ (4)

The updated parameters of PWLCM-3 are calculated by Eq (5) as:


$$\begin{array}{c} \left\{ \begin{array}{c} x\left(1\right)=x_{1}-\left(\begin{array}{c} \left(\frac{{HX}_{25}+{HX}_{26}+\cdots +{HX}_{30}}{10^{15}}\right)-\\ \left\lceil \frac{{HX}_{25}+{HX}_{26}+\cdots +{HX}_{30}}{10^{15}}\right\rceil \end{array}\;\right)\times 0.01\\ cc=c_{1}-\left(\begin{array}{c} \left(\frac{{HX}_{31}+{HX}_{32}+\cdots +{HX}_{36}}{10^{15}}\right)-\\ \left\lceil \frac{{HX}_{31}+{HX}_{32}+\cdots +{HX}_{36}}{10^{15}}\right\rceil \end{array}\;\right)\times 0.01 \end{array}\right. \end{array}$$ (5)

The updated parameters of PWLCM-4 are obtained by Eq (6) as:


$$\begin{array}{c} \left\{ \begin{array}{c} y\left(1\right)=y_{1}-\left(\begin{array}{c} \left(\frac{{HX}_{37}+{HX}_{38}+\cdots +{HX}_{42}}{10^{15}}\right)-\\ \left\lceil \frac{{HX}_{37}+{HX}_{38}+\cdots +{HX}_{42}}{10^{15}}\right\rceil \end{array}\;\right)\times 0.01\\ dd=d_{1}-\left(\begin{array}{c} \left(\frac{{HX}_{43}+{HX}_{44}+\cdots +{HX}_{48}}{10^{15}}\right)-\\ \left\lceil \frac{{HX}_{43}+{HX}_{44}+\cdots +{HX}_{48}}{10^{15}}\right\rceil \end{array}\;\right)\times 0.01 \end{array}\right. \end{array}$$ (6)

Finally, the updated parameters of PWLCM-5 are computed by Eq (7) as:


$$\begin{array}{c} \left\{ \begin{array}{c} z\left(1\right)=z_{1}-\left(\begin{array}{c} \left(\frac{{HX}_{49}+{HX}_{50}+\cdots +{HX}_{56}}{10^{15}}\right)-\\ \left\lceil \frac{{HX}_{49}+{HX}_{50}+\cdots +{HX}_{56}}{10^{15}}\right\rceil \end{array}\;\right)\times 0.01\\ ee=e_{1}-\left(\begin{array}{c} \left(\frac{{HX}_{57}+{HX}_{58}+\cdots +{HX}_{64}}{10^{15}}\right)-\\ \left\lceil \frac{{HX}_{57}+{HX}_{58}+\cdots +{HX}_{64}}{10^{15}}\right\rceil \end{array}\;\right)\times 0.01 \end{array}\right. \end{array}$$ (7)

where $\left(v_{1},a_{1}\right)$, $\;\left(w_{1},b_{1}\right)$, $\;\left(x_{1},c_{1}\right)$, $\;\left(y_{1},d_{1}\right)$, and $\left(z_{1},e_{1}\right)$ represent the original initial conditions and control parameters of PWLCM-1 to PWLCM-5, respectively. (v(1),aa), (w(1),bb), (x(1),cc), (y(1),dd), and (z(1),ee) denote the updated key parameters. The operator ⌈.⌉ represents the ceiling function.

Parameters obtained by the above method are used as input parameters in PWLCM systems. The obtained parameters ensure the generation of good quality pseudo-random sequences which determine the permutation and diffusion operations in the encryption scheme.

### Encryption

**Fig 1:**
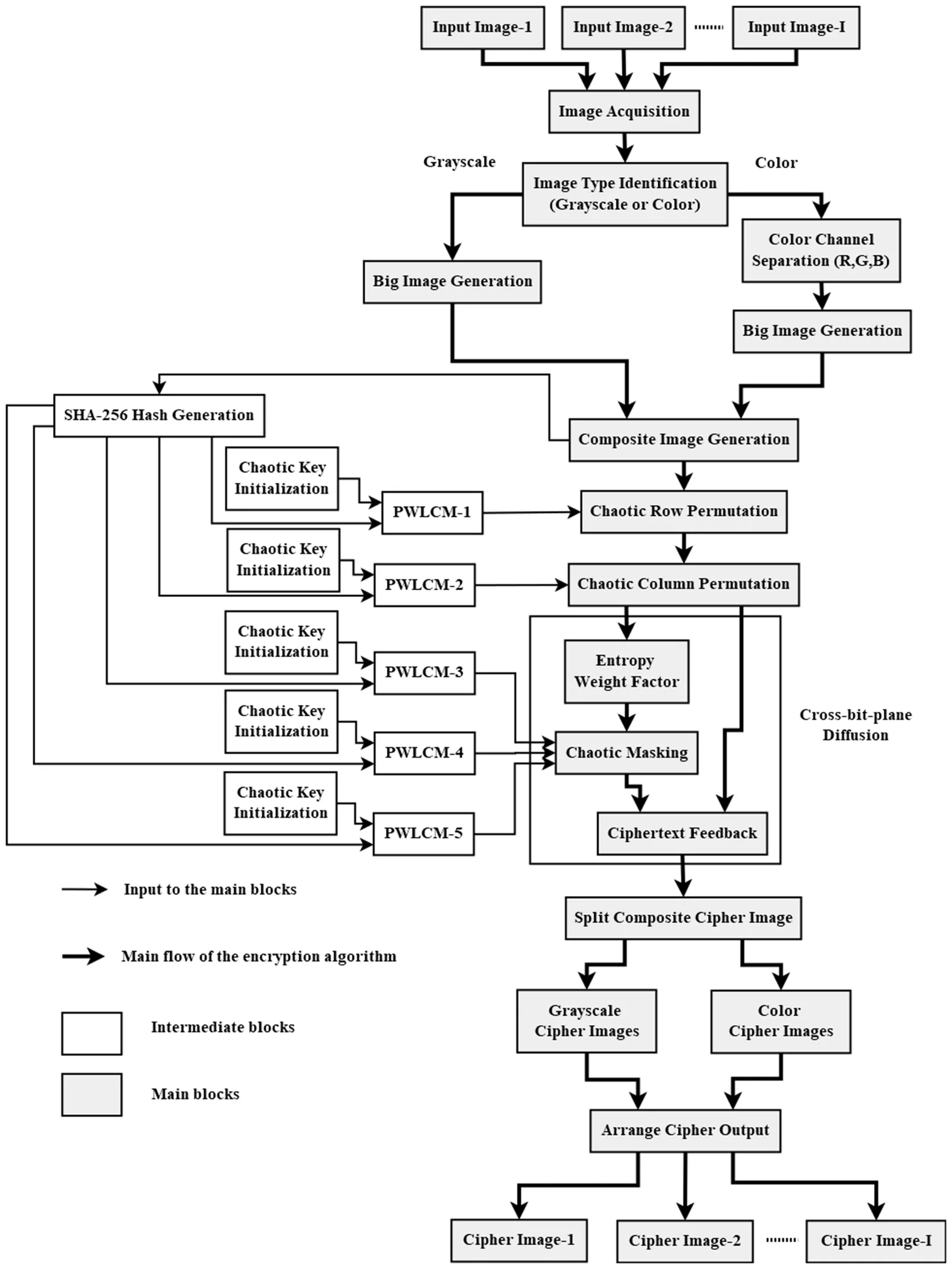
Block diagram for ECPD-MIE encryption scheme.

The encryption process for the proposed ECPD-MIE scheme involves adaptive image concatenation, chaos-based permutation, entropy-driven masking, and cross bit plane diffusion. The complete encryption process is shown in Fig 1 below.

#### 1. Input image processing and composite formation.

Consider a set of *I* input images consisting of *P* grayscale and *Q* color images.

All *P* grayscale images are resized (if required) and horizontally concatenated to form:


$$\begin{array}{c} HVG\in R^{M_{g}\times N_{g}} \end{array}$$

where $M_{g}$ and $N_{g}$ represent the height and width of the concatenated grayscale image, respectively.

Each color image is decomposed into its *RGB* components:


$$\begin{array}{c} R_{i},G_{i},B_{i},i=1,2,...,Q \end{array}$$

The components are concatenated as:


$$\begin{array}{c} HR=\left[R_{1},R_{2},...,R_{Q}\right] \end{array}$$

$$\begin{array}{c} HG=\left[G_{1},G_{2},...,G_{Q}\right] \end{array}$$

$$\begin{array}{c} HB=\left[B_{1},B_{2},...,B_{Q}\right] \end{array}$$

Then, the composite color image is formed as:


$$\begin{array}{c} HVC=\left[\begin{array}{c} HR\\ HG\\ HB \end{array}\right]\in R^{M_{c}\times N_{c}} \end{array}$$

where $M_{c}$ and $N_{c}$ denote the dimensions of the concatenated color image.

Finally, the unified composite image is constructed as:


$$\begin{array}{c} IGC=\left[\begin{array}{c} HVG\\ HVC \end{array}\right]\in R^{M\times N} \end{array}$$

where *M* and *N* denote the dimensions of the unified composite image.

During the composite-image formation stage, the parameters *maxH*, *maxW*, orig_Wg, orig_Wc, gray_sizes, and color_sizes are maintained as auxiliary reconstruction metadata. These parameters record the dimensions of the grayscale and color image groups as well as the original dimensions of each individual image. The reconstruction metadata is subsequently used during decryption to accurately separate the recovered composite image and restore the original image set.

#### 2. Chaotic row permutation.

Using PWLCM-1, generate a chaotic sequence:


$$\begin{array}{c} v=\left\{v\left(1\right),v\left(2\right),\cdots ,v\left(M\right)\right\} \end{array}$$

The sequence is discretized as:


$$\begin{array}{c} v\left(i\right)=\mathrm{mod}\left(\left\lfloor v\left(i\right)\times 10^{6}\right\rfloor ,N\right)+1 \end{array}$$

where ⌊·⌋ denotes the floor operation.

The row permutation is performed as


$$\begin{array}{c} CRP\left(i,:\right)=\mathrm{circshift}\left(IGC\left(i,:\right),\left[0,v\left(i\right)\right]\right),\;i=1,2,...,M. \end{array}$$

The sequence *v* is generated from PWLCM-1 using SHA-256-perturbed chaotic parameters, thereby providing plaintext-dependent row permutation patterns.

#### 3. Chaotic column permutation.

Using PWLCM-2, generate


$$\begin{array}{c} w=\left\{w\left(1\right),w\left(2\right),\cdots ,w\left(N\right)\right\}. \end{array}$$

The sequence is discretized as


$$\begin{array}{c} w\left(j\right)=\mathrm{mod}\left(\left\lfloor w\left(j\right)\times 10^{6}\right\rfloor ,M\right)+1. \end{array}$$

The column permutation is performed as


$$\begin{array}{c} CCP\left(:,j\right)=\mathrm{circshift}\left(CRP\left(:,j\right),\left[w\left(j\right),0\right]\right),\;j=1,2,...,N. \end{array}$$

Although the discretization process may generate repeated shift values, the row and column permutation sequences are generated independently using PWLCM-1 and PWLCM-2, respectively. The successive application of row-wise and column-wise circular shifts provides a strong two-dimensional scrambling effect that substantially rearranges pixel positions over the composite image. Moreover, the SHA-256-based perturbation mechanism dynamically modifies the initial conditions and control parameters of the PWLCM systems for different plaintext images, resulting in distinct permutation patterns for different image sets. Consequently, this design reduces the likelihood of persistent fixed-point structures or short-cycle artifacts that may arise when *M* or *N* are not coprime to the sequence period.

#### 4. Generation of diffusion sequences.

Using PWLCM-3, PWLCM-4, and PWLCM-5, three chaotic diffusion sequences are generated:


$$\begin{array}{c} x,y,z\in R^{M\times N} \end{array}$$

The chaotic matrices are transformed into 8-bit diffusion sequences as follows:


$$\begin{array}{c} x(i,j)=\mathrm{mod}\left(\left\lfloor x(i,j)\times 10^{6}\right\rfloor ,256\right) \end{array}$$

$$\begin{array}{c} y(i,j)=\mathrm{mod}\left(\left\lfloor y(i,j)\times 10^{6}\right\rfloor ,256\right) \end{array}$$

$$\begin{array}{c} z(i,j)=\mathrm{mod}\left(\left\lfloor z(i,j)\times 10^{6}\right\rfloor ,256\right) \end{array}$$

These diffusion sequences are plaintext-dependent since the initial conditions and control parameters of the PWLCM systems are dynamically perturbed using the SHA-256 digest of the composite image.

#### 5. Entropy-controlled weight computation.

The Shannon entropy of the permuted image is computed as


$$\begin{array}{c} EW=-\sum_{k=0}^{255}P(k)\log_{2}P(k) \end{array}$$

where *P*(*k*) denotes the probability of occurrence of the gray level *k*.

The entropy-based weight factor is then obtained as


$$\begin{array}{c} WF=\mathrm{mod}(EW\times 100,256) \end{array}$$

The weight factor *WF* serves as a plaintext-dependent adaptive diffusion parameter that introduces image-dependent variability into the diffusion process. It is not treated as an independent secret-key component; rather, it complements the chaotic diffusion sequences generated by the PWLCM systems.

#### 6. Chaotic masking.

The entropy-based weight factor is incorporated into the chaotic diffusion sequences using the XOR operation:


$$\begin{array}{c} x_{CM}(i,j)=x(i,j)⊕WF \end{array}$$

$$\begin{array}{c} y_{CM}(i,j)=y(i,j)⊕WF \end{array}$$

$$\begin{array}{c} z_{CM}(i,j)=z(i,j)⊕WF \end{array}$$

thereby producing three adaptive diffusion masks.

#### 7. Cross-bit-plane diffusion.

The final cipher image is obtained by combining the three adaptive chaotic masks using XOR operations:


$$\begin{array}{c} CCP(i,j)=CCP(i,j)⊕x_{CM}(i,j)⊕y_{CM}(i,j)⊕z_{CM}(i,j) \end{array}$$

for all i=1,2,...,M and j=1,2,...,N.

Since the XOR operation satisfies both the commutative and associative properties, the ordering of the adaptive chaotic masks does not affect the final ciphertext. Therefore, the sequence $x_{CM}\to y_{CM}\to z_{CM}$ is adopted for implementation consistency and to maintain a direct correspondence with the chaotic sequences generated by PWLCM-3, PWLCM-4, and PWLCM-5, respectively.

The security of the diffusion stage primarily originates from the plaintext-dependent chaotic masks generated by PWLCM-3, PWLCM-4, and PWLCM-5. These adaptive chaotic masks are incorporated into the diffusion process through XOR operations, while the entropy-based weight factor serves as a plaintext-dependent adaptive diffusion parameter that introduces additional image-dependent variability. Consequently, the security contribution of the diffusion stage is mainly derived from the randomness and plaintext sensitivity of the chaotic masks rather than from the ordering of the XOR operations or the entropy-based weight factor alone.

#### 8. Cipher image separation.

The final encrypted composite image is divided to obtain individual cipher images.

To ensure accurate reconstruction during decryption, the auxiliary reconstruction metadata consisting of *maxH*, *maxW*, orig_Wg, orig_Wc, gray_sizes, and color_sizes is maintained together with the encryption parameters. These parameters preserve the partition information of the grayscale and color image groups as well as the original dimensions of all images.

The grayscale portion is horizontally segmented into *P* grayscale cipher images, while the color portion is separated into *CR*, *CG*, and *CB* components and subsequently partitioned and recombined to reconstruct *Q* color cipher images.

Thus, the encrypted image set is given by


$$\begin{array}{c} \left(C_{1},C_{2},\cdots ,C_{I}\right), \end{array}$$

where the associated reconstruction metadata enables deterministic and unambiguous recovery of the original heterogeneous image set during decryption.

It should be noted that the proposed scheme does not require the receiver to infer the number, type, or dimensions of the encrypted images directly from the ciphertext. Instead, the reconstruction metadata maintained during the encryption stage is used to accurately recover the grayscale and color image groups and restore the original image dimensions during decryption.

### Decryption

The decryption process of the proposed ECPD-MIE scheme is the exact inverse of the encryption procedure. Using the same secret chaotic parameters, the SHA-256-based plaintext-dependent perturbation mechanism, the entropy-based adaptive diffusion parameter, and the auxiliary reconstruction metadata, the chaotic permutation sequences and diffusion masks generated during encryption are accurately reproduced. Since the permutation and diffusion operations are reversible, the original plaintext images can be recovered without information loss. The detailed decryption steps are described as follows.

#### 1. Cipher image reception.

The receiver obtains the encrypted composite image together with the secret chaotic parameters of the five PWLCM systems, namely $\left(v_{1},a_{1}\right)$, $\left(w_{1},b_{1}\right)$, $\left(x_{1},c_{1}\right)$, $\left(y_{1},d_{1}\right)$, and $\left(z_{1},e_{1}\right)$, as well as the auxiliary reconstruction metadata required for image recovery. The reconstruction metadata includes the composite image dimensions, the original widths of the grayscale and color image groups, and the size information of each grayscale and color image. Using the same composite image, the SHA-256 digest is regenerated and employed as a plaintext-dependent perturbation mechanism to reproduce the chaotic initial conditions and control parameters used during encryption. In addition, the entropy-based weight factor is recomputed to reconstruct the adaptive diffusion masks.

#### 2. Cipher image concatenation.

The received cipher images are first categorized into grayscale and color images.

All grayscale cipher images are horizontally concatenated to form the composite grayscale image:


$$\begin{array}{c} HVG\in R^{M_{g}\times N_{g}} \end{array}$$

Similarly, the color cipher images are combined to form the composite color image:


$$\begin{array}{c} HVC\in R^{M_{c}\times N_{c}} \end{array}$$

To maintain consistency with the encryption stage, both components are merged to reconstruct the unified composite cipher image:


$$\begin{array}{c} IGC=\left[\begin{array}{c} HVG\\ HVC \end{array}\right]\in R^{M\times N} \end{array}$$

#### 3. Regeneration of chaotic diffusion sequences.

Using the regenerated PWLCM-3, PWLCM-4, and PWLCM-5 parameters, each chaotic system is iterated M×N times to generate the diffusion sequences


$$\begin{array}{c} x,y,z\in R^{M\times N}. \end{array}$$

These sequences are transformed into 8-bit diffusion matrices as follows:


$$\begin{array}{c} x(i,j)=\mathrm{mod}\left(\left\lfloor x(i,j)\times 10^{6}\right\rfloor ,256\right) \end{array}$$

$$\begin{array}{c} y(i,j)=\mathrm{mod}\left(\left\lfloor y(i,j)\times 10^{6}\right\rfloor ,256\right) \end{array}$$

$$\begin{array}{c} z(i,j)=\mathrm{mod}\left(\left\lfloor z(i,j)\times 10^{6}\right\rfloor ,256\right) \end{array}$$

The generated diffusion sequences remain plaintext-dependent because the PWLCM parameters are dynamically perturbed using the SHA-256 digest of the composite image.

#### 4. Entropy weight factor computation.

The entropy-based weight factor is regenerated as


$$\begin{array}{c} WF=\mathrm{mod}(EW\times 100,256), \end{array}$$

where *EW* denotes the Shannon entropy of the permuted image.

The parameter *WF* serves as a plaintext-dependent adaptive diffusion parameter and is used to reproduce the same diffusion masks generated during the encryption stage. It is not treated as an independent secret-key component.

#### 5. Adaptive chaotic mask generation.

The entropy-based weight factor is incorporated into the chaotic diffusion sequences to regenerate the adaptive diffusion masks:


$$\begin{array}{c} x_{CM}(i,j)=x(i,j)⊕WF \end{array}$$

$$\begin{array}{c} y_{CM}(i,j)=y(i,j)⊕WF \end{array}$$

$$\begin{array}{c} z_{CM}(i,j)=z(i,j)⊕WF \end{array}$$

These masks are identical to those used during encryption and are subsequently employed for the reverse diffusion process.

#### 6. Reverse cross-bit-plane diffusion.

The inverse diffusion process is performed using the regenerated adaptive chaotic masks. Since the XOR operation is self-invertible, associative, and commutative, the original permuted image can be accurately recovered by applying the same diffusion masks generated during the encryption stage.


$$\begin{array}{c} IGC(i,j)=IGC(i,j)⊕z_{CM}(i,j)⊕y_{CM}(i,j)⊕x_{CM}(i,j) \end{array}$$

for all i=1,2,...,M and j=1,2,...,N.

Owing to the associative and commutative properties of the XOR operation, any permutation of the adaptive chaotic masks yields the same recovery result. The ordering $z_{CM}\to y_{CM}\to x_{CM}$ is retained solely for implementation consistency with the decryption procedure.

#### 7. Reverse chaotic column permutation.

Using PWLCM-2, the chaotic sequence *w* is regenerated and discretized as


$$\begin{array}{c} w\left(j\right)=\mathrm{mod}\left(\left\lfloor w\left(j\right)\times 10^{6}\right\rfloor ,M\right)+1. \end{array}$$

The inverse column permutation is performed as


$$\begin{array}{c} ICRP\left(:,j\right)=\mathrm{circshift}\left(IGC\left(:,j\right),\left[-w\left(j\right),0\right]\right),\;j=1,2,...,N. \end{array}$$

The same plaintext-dependent chaotic sequence generated during encryption is reproduced, ensuring accurate recovery of the column permutation stage.

#### 8. Reverse chaotic row permutation.

Using PWLCM-1, the chaotic sequence *v* is regenerated and discretized as


$$\begin{array}{c} v\left(i\right)=\mathrm{mod}\left(\left\lfloor v\left(i\right)\times 10^{6}\right\rfloor ,N\right)+1. \end{array}$$

The inverse row permutation is performed as


$$\begin{array}{c} IP\left(i,:\right)=\mathrm{circshift}\left(ICRP\left(i,:\right),\left[0,-v\left(i\right)\right]\right),\;i=1,2,...,M. \end{array}$$

Since the same SHA-256-perturbed PWLCM parameters are regenerated during decryption, the original row permutation sequence is reproduced exactly, enabling lossless recovery of the plaintext image.

#### 9. Recovery of original images.

After inverse diffusion and inverse permutation, the recovered composite image is separated into grayscale and color components using the auxiliary reconstruction metadata. Specifically, the parameters *maxH*, *maxW*, orig_Wg, orig_Wc, gray_sizes, and color_sizes are used to determine the boundaries of the grayscale and color image groups and to restore the original dimensions of each image.

The grayscale component is recovered as


$$\begin{array}{c} HVG=IP(1:maxH,1:orig\_Wg), \end{array}$$

while the color component is recovered as


$$\begin{array}{c} HVC=IP(maxH+1:end,1:orig\_Wc). \end{array}$$

The grayscale component is then partitioned according to the entries in gray_sizes, whereas the color component is first separated into the red, green, and blue channels and subsequently partitioned according to the entries in color_sizes. The corresponding RGB channels are finally recombined to recover the original color images.

Thus, the recovered image set is given by


$$\begin{array}{c} (D_{1},D_{2},\cdots ,D_{I}), \end{array}$$

which exactly corresponds to the original input images, thereby confirming the correctness, deterministic reconstruction capability, lossless recovery, and reversibility of the proposed ECPD-MIE cryptosystem.

It should be noted that the proposed scheme does not require the receiver to infer the number, type, or dimensions of the encrypted images directly from the ciphertext. Instead, the reconstruction metadata consisting of *maxH*, *maxW*, orig_Wg, orig_Wc, gray_sizes, and color_sizes is maintained together with the decryption parameters. This metadata enables deterministic and unambiguous separation of the grayscale and color image groups and accurate recovery of all individual images.

### Simulation results and security analysis

**Fig 2:**
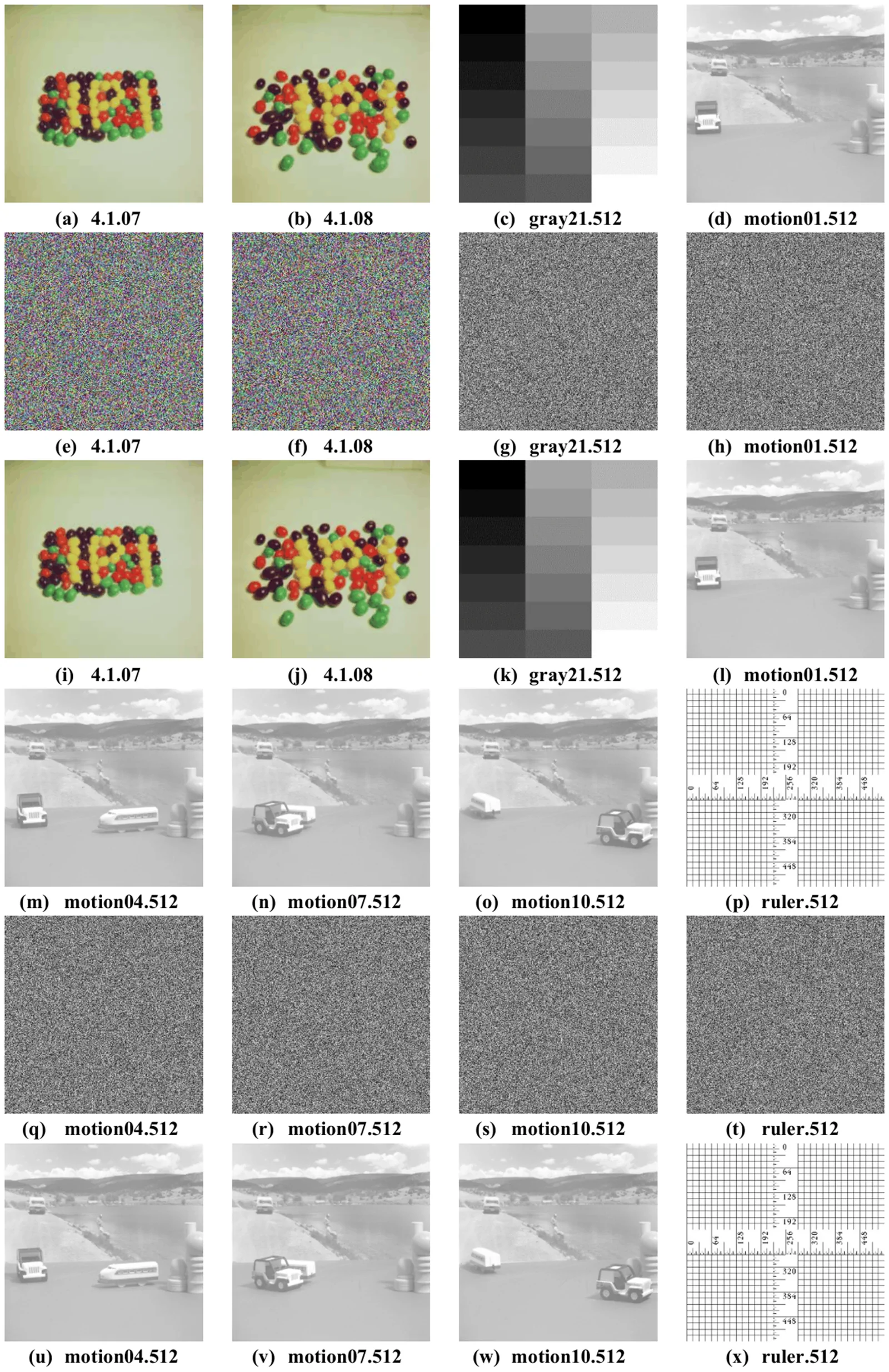
Simulation results: (a,b) original color images, (e,f) encrypted color images, (i,j) decrypted color images, (c,d,m–p) original grayscale images, (g,h,q–t) encrypted grayscale images, (k,l,u–x) decrypted grayscale images.

Simulation results for the proposed ECPD-MIE scheme demonstrate its effectiveness in securely encrypting heterogeneous image sets containing both grayscale and color images within a unified framework. Fig 2 presents the overall encryption and decryption results obtained using the proposed scheme, while the detailed performance and security analyses are discussed in the subsequent sections.

To evaluate the performance of the proposed algorithm, experiments were conducted using eight standard benchmark images obtained from the USC-SIPI Image Database [43]. The experimental image set consists of six grayscale images of size 512×512, namely “gray21.512.tiff”, “motion01.512.tiff”, “motion04.512.tiff”, “motion07.512.tiff”, “motion10.512.tiff”, and “ruler.512.tiff”, together with two color images of size 256×256, namely “4.1.07.tiff” and “4.1.08.tiff”. The selected images exhibit diverse visual characteristics, including natural scenes, textured regions, motion patterns, and geometric structures, making them suitable for evaluating the effectiveness of heterogeneous multi-image encryption algorithms. Unlike conventional image encryption schemes that generally assume homogeneous image sets, the proposed ECPD-MIE framework simultaneously processes grayscale and color images of different dimensions by first constructing a unified composite image, thereby demonstrating its flexibility for practical multimedia applications.

It is observed that the security of the proposed encryption scheme depends on multiple interacting components, including the initial conditions and control parameters of the five PWLCM systems, the SHA-256 digest of the composite image, and the entropy-based weight factor obtained from the permuted image. In the proposed framework, the SHA-256 hash is employed as a plaintext-dependent perturbation mechanism rather than as an independent secret key. The generated hash dynamically modifies the initial conditions and control parameters of the PWLCM systems, thereby ensuring strong plaintext sensitivity. Consequently, even a slight variation in the input image set produces different chaotic sequences for permutation and diffusion. Together, these components contribute to the generation of highly random chaotic sequences and enhance the resistance of the proposed cryptosystem against brute-force, statistical, differential, and known-plaintext attacks. The parameters used in the experiments are listed in Table 1.

**Table 1:** Secret key parameters and plaintext-dependent security parameters.

Component		Control parameters	Initial values
PWLCM Chaotic Systems	1	*a*_1_ = 0.379018545523799	*v*_1_ = 0.259750719928723
	2	*b*_1_ = 0.387429913321760	*w*_1_ = 0.259813765422011
	3	*c*_1_ = 0.379014327853663	*x*_1_ = 0.259467125378536
	4	*d*_1_ = 0.385710542756184	*y*_1_ = 0.264027834658214
	5	*e*_1_ = 0.370259761354639	*z*_1_ = 0.264978136720841
SHA-256 Digest		Plaintext-dependent perturbation used to modify	
		PWLCM initial conditions and control parameters	
Entropy-based Weight Factor (*WF*)		Plaintext-dependent adaptive diffusion parameter	
		computed from the entropy of the permuted image	

Fig 2 illustrates the visual outcomes of the entire encryption and decryption procedure. The original grayscale and color images are transformed into noise-like encrypted images without revealing any perceptual information. During the decryption process, all original images are accurately reconstructed without any observable distortion, thereby demonstrating the correctness and reversibility of the proposed cryptosystem.

An inherent advantage of the proposed ECPD-MIE framework is its capability to simultaneously process grayscale and color images of different dimensions through a unified composite-image architecture. This design improves computational efficiency while preserving the visual characteristics of both image types and eliminates the need for separate encryption procedures for heterogeneous image sets.

Overall, the proposed cryptosystem demonstrates excellent security performance, high reconstruction quality, and efficient heterogeneous multi-image encryption capability. To further validate the security and robustness of the proposed framework, comprehensive security analyses are presented in the following subsections.

### Key space analysis

Key space analysis is one of the important criteria used to evaluate the resistance of an encryption algorithm against brute-force attacks. A sufficiently large key space increases the computational complexity required for an attacker to exhaustively search all possible secret keys. In modern cryptographic systems, a key space larger than 2^128^ is generally considered secure against brute-force attacks [44,45].

In the proposed ECPD-MIE scheme, the primary secret key consists of the initial conditions and control parameters of the five PWLCM chaotic systems, namely $\left(v_{1},a_{1},w_{1},b_{1},x_{1},c_{1},y_{1},d_{1},z_{1},e_{1}\right)$. Each parameter is represented using double-precision floating-point arithmetic with an effective precision of approximately 10^15^ [46]. Therefore, the key space associated with the chaotic parameters can be estimated as


$$\begin{array}{c} {\left(10^{15}\right)}^{10}=10^{150}. \end{array}$$

Using the relation $\log_{2}(10^{150})\approx 498.29$, the corresponding key space can be expressed as


$$\begin{array}{c} 10^{150}\approx 2^{498.29}. \end{array}$$

In addition to the secret chaotic parameters, the proposed scheme incorporates a SHA-256 hash of the composite image and an entropy-based weight factor. It should be noted that these two components are plaintext-dependent quantities and are not treated as independent secret-key elements. Instead, they dynamically perturb the chaotic systems and diffusion process, thereby enhancing plaintext sensitivity and strengthening resistance against known-plaintext and chosen-plaintext attacks. Consequently, they improve the practical security of the cryptosystem but are not included in the formal key-space computation.

Therefore, the effective secret-key space of the proposed ECPD-MIE scheme is approximately


$$\begin{array}{c} 10^{150}\approx 2^{498.29}, \end{array}$$

which is substantially larger than the minimum security requirement of 2^128^ and provides strong resistance against exhaustive brute-force attacks.

Table 2 presents the detailed key-space evaluation of the proposed ECPD-MIE scheme, while Table 3 compares the key space of the proposed method with several recently reported multi-image encryption algorithms [15–21,25–40]. The comparison demonstrates that the proposed scheme provides a sufficiently large key space for resisting brute-force attacks. Furthermore, the incorporation of plaintext-dependent SHA-256-based chaotic perturbation and entropy-controlled diffusion enhances the plaintext sensitivity and practical security of the proposed cryptosystem.

**Table 2:** Key space analysis.

Component		Key Space	Contribution
PWLCM Chaotic Systems	1	$\left(v_{1}\to 10^{15}\right),\;\left(a_{1}\to 10^{15}\right)$	${\left(10^{15}\right)}^{10}$ $\approx 10^{150}$ $\approx 2^{498.29}$
	2	$\left(w_{1}\to 10^{15}\right),\;\left(b_{1}\to 10^{15}\right)$	
	3	$\left(x_{1}\to 10^{15}\right),\;\left(c_{1}\to 10^{15}\right)$	
	4	$\left(y_{1}\to 10^{15}\right),\;\left(d_{1}\to 10^{15}\right)$	
	5	$\left(z_{1}\to 10^{15}\right),\;\left(e_{1}\to 10^{15}\right)$	
SHA-256 Hash Value		256-bit digest	Plaintext-dependent perturbation
Entropy-based Weight Factor		8-bit adaptive parameter	Plaintext-dependent diffusion control
**Effective Secret Key Space**			$10^{150}\approx 2^{498.29}$

**Table 3:** Comparison of total key space.

Algorithms	Total Key Space
[15]	$7.3724\times 10^{134}\approx 8.1148\times 2^{445}$
[16]	$10^{56}\approx 1.0196\times 2^{186}$
[17]	$10^{60}\approx 1.2446\times 2^{199}$
[18]	$1.964\times 2^{426}$
[19]	$10^{56}\approx 1.0196\times 2^{186}$
[20]	$1.5491\times 2^{526}$
[21]	$10^{141}\approx 1.3121\times 2^{468}$
[25]	$9.4599\times 10^{76}\approx 13.0717\times 2^{252}$
[26]	$1.1579\times 10^{189}\approx 2^{628}$
[27]	${10^{249}\approx 2}^{827}$
[28]	2^332^
[29]	2^555^
[30]	${10^{87}\approx 2}^{289}$
[31]	>2^512^
[32]	2^256^
[33]	${10^{142}\approx 2}^{472}$
[34]	2^476^
[35]	2^10524^
[36]	>2^400^
[37]	${{\left(10^{15}\right)}^{17}\approx 2}^{1359}$
[38]	2^519^
[39]	${10^{182}\approx 2}^{605}$
[40]	2^256^
**Proposed**	$10^{150}\approx 2^{498.29}$

### Histogram analysis

Histogram analysis is an important statistical tool for evaluating the resistance of image encryption algorithms against statistical attacks. A secure image encryption scheme should produce encrypted images whose histograms are significantly different from those of the corresponding plaintext images and exhibit an approximately uniform distribution [47,48]. In the proposed study, histogram analysis is performed for both grayscale and color images. For color images, the histograms of the red, green, and blue channels are analyzed simultaneously to provide a comprehensive assessment of the statistical characteristics of the encrypted images.

**Fig 3:**
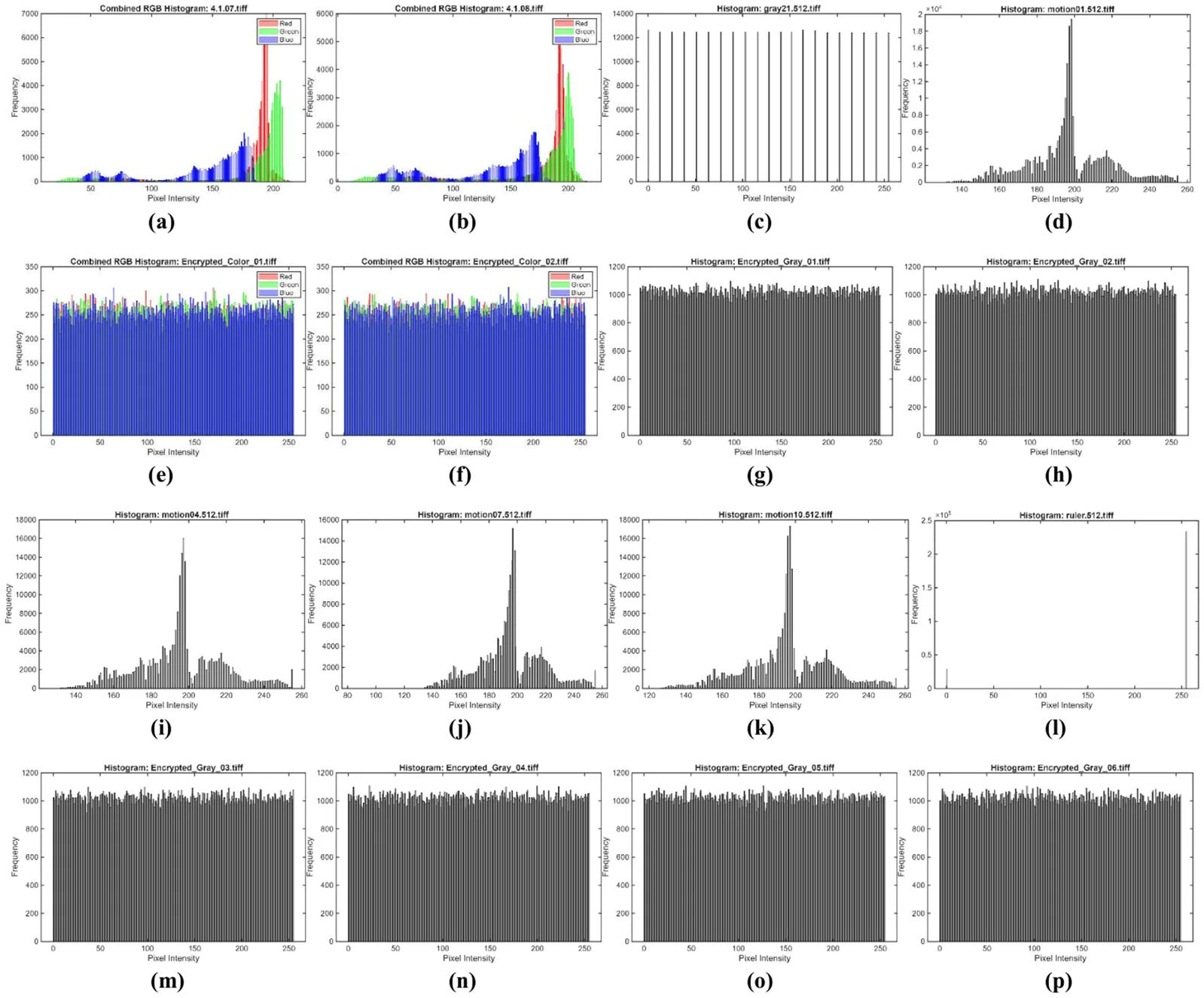
Histogram analysis results: (a,b) original color images, (e,f) encrypted color images, (c,d,i–l) original grayscale images, and (g,h,m–p) encrypted grayscale images.

Fig 3 presents the histogram distributions of representative grayscale and color images before and after encryption. The plaintext images exhibit highly non-uniform histogram distributions that reflect the inherent statistical characteristics of the image contents. In contrast, the corresponding encrypted images exhibit nearly uniform histogram distributions without any discernible statistical patterns. For the grayscale images, the encrypted histograms are uniformly distributed across the entire intensity range. Similarly, for the color images, the histograms of the red, green, and blue channels become uniformly distributed after encryption, indicating that the statistical characteristics of the original images have been effectively concealed. These observations demonstrate that the proposed ECPD-MIE scheme successfully removes the statistical information of the plaintext images and provides strong resistance against statistical attacks.

### Histogram variance analysis

Histogram variance is used to evaluate the uniformity of pixel intensity distributions in encrypted images. A lower histogram variance indicates a more uniform distribution, which is desirable for secure image encryption because it reduces statistical leakage and makes statistical attacks more difficult [49,50].

For an image, the histogram variance is defined by Eq. 8 as


$$\begin{array}{c} var(c)=\frac{1}{256}\sum_{k=0}^{255}{\left(c_{k}-\bar{c}\right)}^{2} \end{array}$$ (8)

where $c_{k}$ denotes the number of pixels having intensity level *k*, and $\bar{c}$ is the mean histogram value given by Eq. 9:


$$\begin{array}{c} \bar{c}=\frac{M\times N}{256} \end{array}$$ (9)

where M×N denotes the image size.

For grayscale images, the histogram variance is computed directly from the image histogram. For color images, the histogram variance is calculated independently for the red, green, and blue channels, and the average of the three channel variances is reported as the histogram variance of the color image.

Table 4 presents the histogram variance values of the plaintext and encrypted images. It can be observed that the encrypted images exhibit significantly lower histogram variance values than their corresponding plaintext images, indicating a much more uniform distribution of pixel intensities. This demonstrates that the proposed ECPD-MIE scheme effectively removes the statistical characteristics of the original images and significantly reduces statistical leakage.

**Table 4:** Histogram variance analysis.

Types of Images	Images	Original	Encrypted
Color	4.1.07	486579.0911	253.9609
	4.1.08	338837.9115	241.4141
Grayscale	gray21.512	11734454.8594	863.0234
	motion01.512	5277402.4844	1051.9531
	motion04.512	4327485.0938	1004.7500
	motion07.512	4151409.1172	1023.6875
	motion10.512	4597947.7188	1013.8359
	ruler.512	214810988.5313	1080.4766

### Chi-square test analysis

The chi-square test is widely used to evaluate the statistical uniformity of the pixel intensity distribution in encrypted images. It measures the deviation between the observed histogram distribution and the ideal uniform distribution. For a secure image encryption algorithm, the histogram of the encrypted image should closely follow a uniform distribution, resulting in a relatively small chi-square statistic [51,52].

The chi-square statistic is computed using Eq. 10:


$$\begin{array}{c} \chi^{2}=\sum_{j=0}^{255}\frac{{\left(of_{j}-ef_{j}\right)}^{2}}{ef_{j}} \end{array}$$ (10)

where $of_{j}$ denotes the observed frequency of intensity level *j*, and the expected frequency is given by Eq. 11:


$$\begin{array}{c} ef_{j}=\frac{M\times N}{256} \end{array}$$ (11)

where M×N denotes the image size.

For the proposed ECPD-MIE scheme, the chi-square test is performed for both grayscale and color images. For color images, the chi-square statistic is computed independently for the red, green, and blue channels, and the average value of the three channels is reported.

The chi-square values of the plaintext and encrypted images are presented in Table 5. It can be observed that the encrypted images exhibit significantly improved histogram uniformity compared with their corresponding plaintext images. Furthermore, Table 6 compares the chi-square values of the proposed method with those reported by representative image encryption algorithms. The comparison shows that the proposed ECPD-MIE scheme achieves chi-square values comparable to those of existing state-of-the-art methods while satisfying the critical values corresponding to the 5% and 1% significance levels. These results demonstrate that the proposed encryption scheme effectively conceals the statistical characteristics of the plaintext images and provides strong resistance against statistical attacks.

**Table 5:** Chi-square test $\left(\chi_{test}^{2}\right)$ analysis.

Types of images	Images	Chi-square test results		Pass/Fail results	
		Original	Encrypted	$\chi_{255,0.05}^{2}=293.2478$	$\chi_{255,0.01}^{2}=310.457$
Color	4.1.07	486579.0911	253.9609	Pass	Pass
	4.1.08	338837.9115	241.4141	Pass	Pass
Grayscale	gray21.512	2933613.7148	215.7559	Pass	Pass
	motion01.512	1319350.6211	262.9883	Pass	Pass
	motion04.512	1081871.2734	251.1875	Pass	Pass
	motion07.512	1037852.2793	255.9219	Pass	Pass
	motion10.512	1149486.9297	253.4590	Pass	Pass
	ruler.512	53702747.1328	270.1191	Pass	Pass
Overall Average		7756292.3692	250.6008	Pass	Pass

**Table 6:** Comparison of $\chi_{test}^{2}$ results of encrypted images.

Algorithms	Average Chi-square test
[20]	243.4365
[27]	258.6456
[28]	254.2
[29]	246.6905
[30]	264.1387
[34]	246.6673
[38]	253.7346
**Proposed**	250.6008

### Pixel correlation analysis

Pixel correlation analysis is employed for evaluating the strength of an encryption technique in reducing the correlations of pixels, which are usually very high in natural images. In plaintext images, it is expected that the correlation of neighboring pixels is very high due to spatial redundancy, but it should be minimized as close to zero as possible using a secure encryption technique [53–55].

The correlation coefficient between two adjacent pixels *u* and *v* is defined by Eq 12 as:


$$\begin{array}{c} {corr}_{uv}=\frac{cov\left(u,v\right)}{\sqrt{D\left(u\right)}\sqrt{D\left(v\right)}} \end{array}$$ (12)

where the covariance and variance are given by Eqs 13–17 as:


$$\begin{array}{c} cov\left(u,v\right)=\frac{1}{P}\sum_{i=1}^{P}\left(u_{i}-E\left(u\right)\right)\left(v_{i}-E\left(v\right)\right) \end{array}$$ (13)

$$\begin{array}{c} D\left(u\right)=\frac{1}{P}\sum_{i=1}^{P}{\left(u_{i}-E\left(u\right)\right)}^{2} \end{array}$$ (14)

$$\begin{array}{c} D\left(v\right)=\frac{1}{P}\sum_{i=1}^{P}{\left(v_{i}-E\left(v\right)\right)}^{2} \end{array}$$ (15)

$$\begin{array}{c} E\left(u\right)=\frac{1}{P}\sum_{i=1}^{P}u_{i} \end{array}$$ (16)

$$\begin{array}{c} E\left(v\right)=\frac{1}{P}\sum_{i=1}^{P}v_{i} \end{array}$$ (17)

where *P* denotes the number of randomly selected pixel pairs. In this study, 10,000-pixel pairs are randomly selected in horizontal, vertical, and diagonal directions to ensure reliable statistical evaluation.

**Fig 4:**
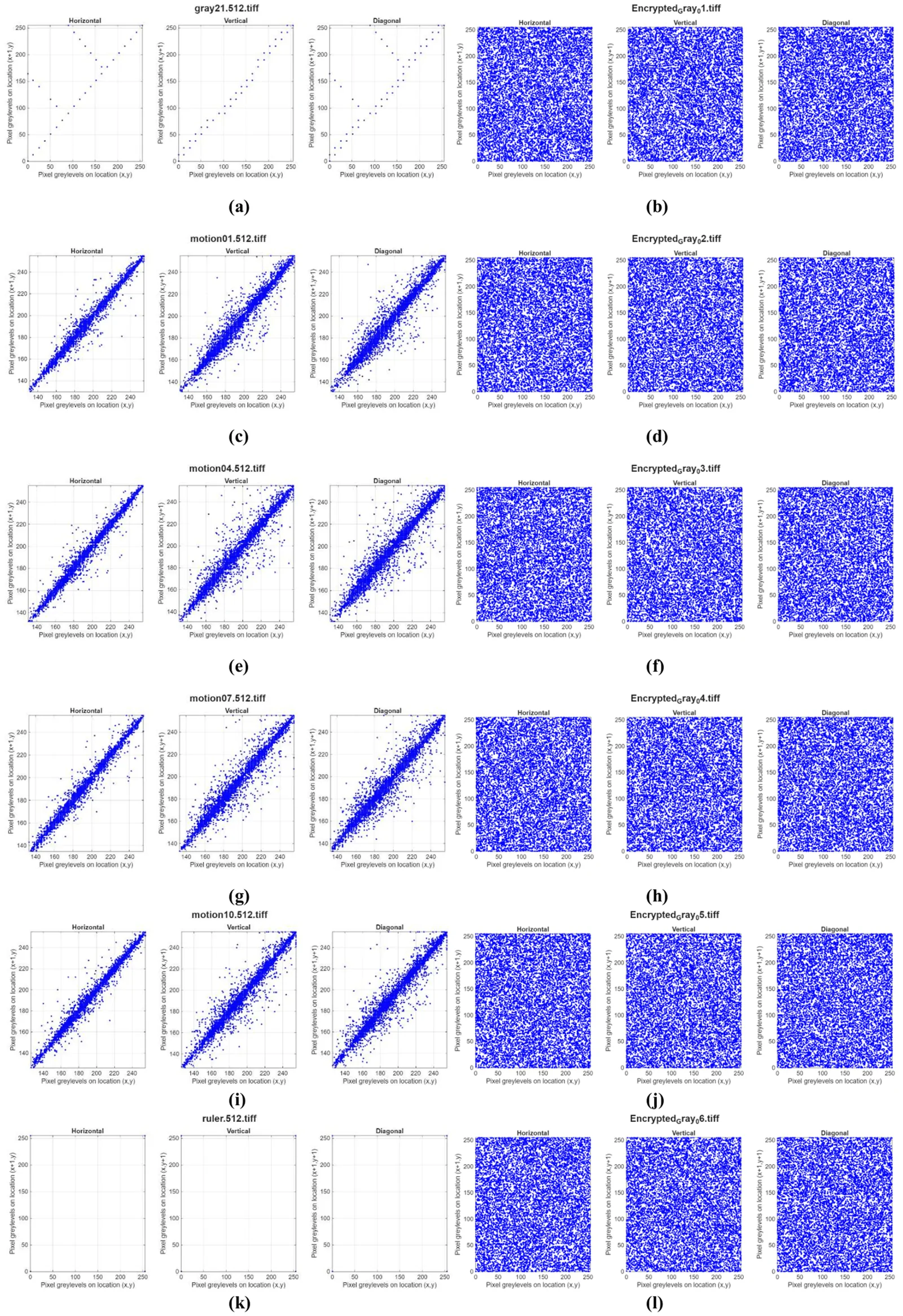
Pixel correlation results of grayscale images: (a,c,e,g,i,k) original images, (b,d,f,h,j,l) encrypted images.

**Fig 5:**
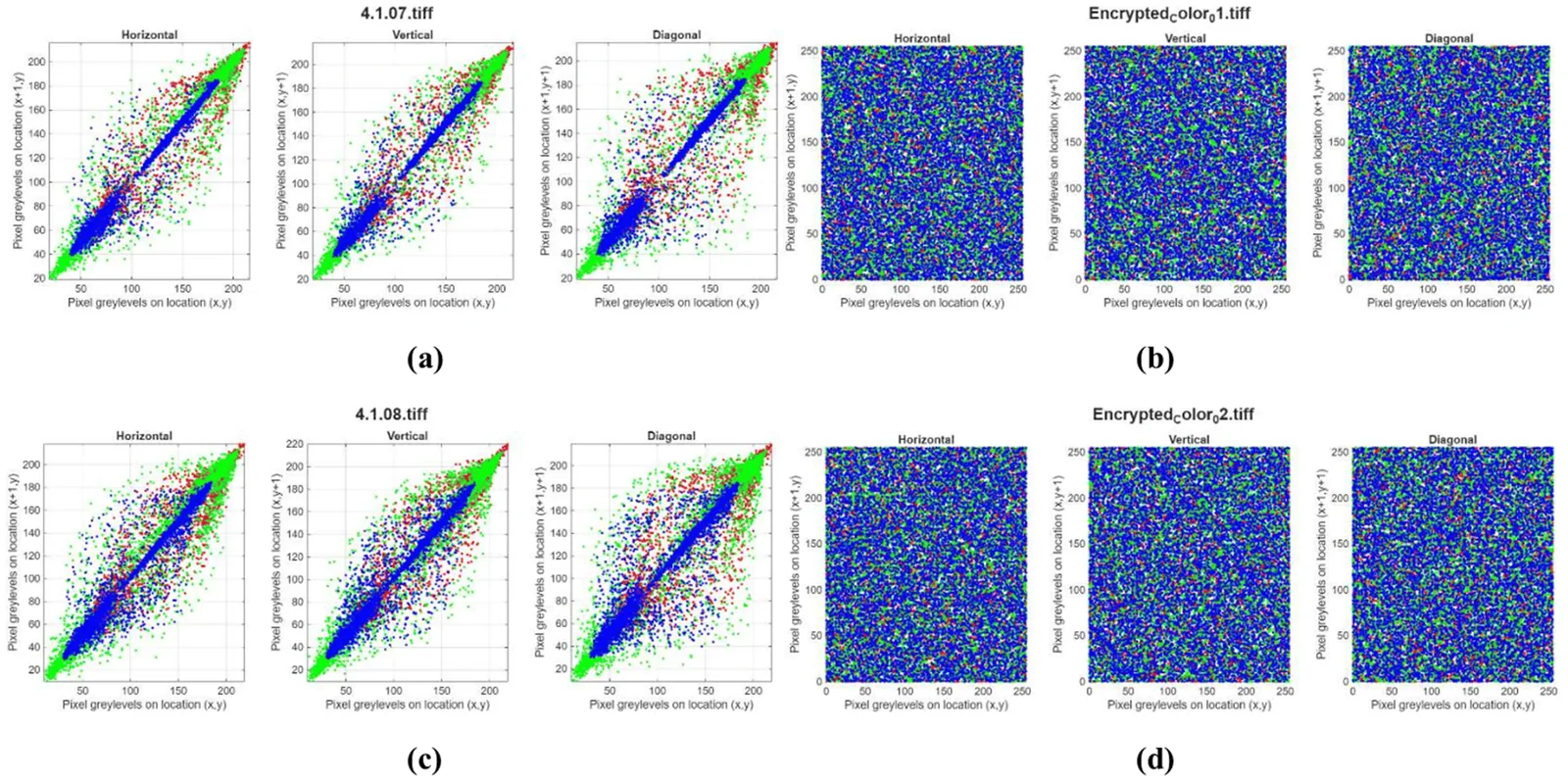
Pixel correlation results of color images: (a,c) original images, (b,d) encrypted images.

Figs 4 and 5 illustrate the correlation distributions of adjacent pixels for representative grayscale and color images, respectively. The plaintext images exhibit strong linear clustering in the horizontal, vertical, and diagonal directions, indicating the high spatial correlation that naturally exists in digital images. In contrast, the encrypted images display uniformly scattered distributions without any discernible linear relationship, indicating that the proposed ECPD-MIE scheme effectively destroys the statistical dependence among neighboring pixels.

The quantitative results presented in Table 7 further confirm this observation. The plaintext images exhibit correlation coefficients close to one in all three directions, whereas the encrypted images exhibit correlation coefficients approaching zero. Furthermore, the comparative results shown in Table 8 demonstrate that the proposed scheme achieves correlation coefficients comparable to or closer to zero than those reported by representative state-of-the-art image encryption algorithms. This excellent decorrelation performance can be attributed to the combined effects of the SHA-256-based plaintext-dependent chaotic perturbation mechanism, the independent PWLCM-based row and column permutation stages, and the entropy-controlled adaptive diffusion process employed in the proposed ECPD-MIE framework.

**Table 7:** Pixel correlation analysis.

Types of Images	Images	Original			Encrypted		
		Horizontal	Vertical	Diagonal	Horizontal	Vertical	Diagonal
Color	4.1.07	0.9788	0.9804	0.9624	0.0013	0.0008	0.0091
	4.1.08	0.9745	0.9760	0.9497	0.0105	0.0126	−0.0023
Grayscale	gray21.512	0.9959	0.9998	0.9958	0.0147	−0.0039	0.0000
	motion01.512	0.9911	0.9797	0.9745	0.0072	−0.0132	0.0072
	motion04.512	0.9918	0.9750	0.9699	−0.0186	−0.0132	−0.0021
	motion07.512	0.9924	0.9785	0.9743	0.0088	−0.0010	−0.0044
	motion10.512	0.9922	0.9796	0.9753	−0.0052	0.0042	0.0130
	ruler.512	0.4536	0.4708	−0.0252	0.0080	−0.0083	−0.0029
Average		0.9213	0.9175	0.8471	0.0033	−0.0028	0.0022

**Table 8:** Comparison of average adjacent pixel correlation of encrypted images.

Algorithms	Horizontal	Vertical	Diagonal
[15]	−0.0299	0.0309	0.0039
[17]	−0.0012	0.0017	0.0041
[18]	−0.0006	−0.0001	−0.0020
[19]	−0.0022	−0.0025	0.0016
[20]	0.0012	−0.0007	−0.0009
[21]	$-1.0069\times 10^{-7}$	$-3.9354\times 10^{-7}$	$-0.7142\times 10^{-7}$
[25]	−0.0036	0.0016	−0.0058
[26]	−0.0003	0.0011	0.0013
[27]	0.0021	0.0029	0.0023
[28]	0.0027	0.0018	0.0002
[29]	0.0011	0.0008	0.0015
[30]	−0.0035	−0.0018	0.0065
[31]	0.0015	0.0011	0.0015
[32]	0.0020	0.0003	0.0005
[33]	0.0040	0.0037	0.0030
[34]	0.0022	0.0020	0.0022
[35]	−0.0008	0.0005	−0.0016
[36]	0.0017	0.0028	−0.0010
[37]	0.0002	−0.0024	0.0006
[38]	0.0002	0.0006	−0.0004
[39]	−0.0022	−0.0014	−0.0005
[40]	−0.0730	−0.1100	−0.0700
**Proposed**	0.0033	−0.0028	0.0022

### MSE, PSNR, and SSIM analysis

Mean Squared Error (MSE), Peak Signal-to-Noise Ratio (PSNR), and Structural Similarity Index Measure (SSIM) are widely used image quality metrics for evaluating the distortion introduced during encryption and the reconstruction quality achieved after decryption. In the proposed ECPD-MIE scheme, these metrics are computed by comparing the original image with both the encrypted and the corresponding decrypted images to assess the effectiveness and reversibility of the encryption process.

The proposed ECPD-MIE framework evaluates these image quality metrics for both grayscale and color images, thereby demonstrating its capability to securely encrypt and accurately reconstruct heterogeneous image sets within a unified encryption framework.

#### Mean Squared Error (MSE).

MSE is a measure of the mean of the squared differences between corresponding pixels in two given images. A higher value of MSE between the original and encrypted images implies that a large amount of distortion is introduced during the encryption process, which is a favorable condition for security [56,57]. Similarly, a zero value of MSE between the original and decrypted images implies a perfect reconstruction [56].

The MSE is defined by Eqs. 18 and 19 as:


$$\begin{array}{c} {MSE}_{OI-EI}=\frac{1}{M\times N}\sum_{k=1}^{M}\sum_{l=1}^{N}{\left({OI}_{kl}-{EI}_{kl}\right)}^{2} \end{array}$$ (18)

$$\begin{array}{c} {MSE}_{OI-DI}=\frac{1}{M\times N}\sum_{k=1}^{M}\sum_{l=1}^{N}{\left({OI}_{kl}-{DI}_{kl}\right)}^{2} \end{array}$$ (19)

where *OI*, *EI*, and *DI* denote the original, encrypted, and decrypted images, respectively, and M×N represents the image size. The measure ${MSE}_{OI-EI}$ calculates the distortion between the original and encrypted images, while ${MSE}_{OI-DI}$ calculates the distortion between the original and decrypted images.

#### Peak Signal-to-Noise Ratio (PSNR).

PSNR is derived from MSE and measures the quality difference between two images. A lower PSNR value between the original and encrypted images indicates a greater difference and stronger encryption. In contrast, for successful decryption, the PSNR value between the original and decrypted images tends toward infinity [56].

The PSNR is computed using Eqs. 20 and 21 as:


$$\begin{array}{c} {PSNR}_{OI-EI}=20\log_{10}\left[\frac{I_{maxI}}{\sqrt{{MSE}_{OI-EI}}}\right] \end{array}$$ (20)

$$\begin{array}{c} {PSNR}_{OI-DI}=20\log_{10}\left[\frac{I_{maxI}}{\sqrt{{MSE}_{OI-DI}}}\right] \end{array}$$ (21)

where $I_{maxI}$ is the maximum pixel value (255 for 8-bit images). ${PSNR}_{OI-EI}$ is the PSNR value between original and encrypted images, and ${PSNR}_{OI-DI}$ is the PSNR value between original and decrypted images.

#### Structural Similarity Index Measure (SSIM).

SSIM evaluates perceptual similarity by considering luminance, contrast, and structural information. For an effective encryption scheme, the SSIM value between the original and encrypted images should be close to zero, indicating no structural similarity, while the SSIM between the original and decrypted images should be close to one, indicating accurate reconstruction.

The SSIM is defined using Eqs. 22 and 23 as:


$$\begin{array}{c} {SSIM}_{OI-EI}=\frac{\left(2\mu_{OI}\mu_{EI}+C_{1})(2\sigma_{OI,EI}+C_{2}\right)}{\left(\mu_{OI}^{2}+\mu_{EI}^{2}+C_{1})(\sigma_{OI}^{2}+\sigma_{EI}^{2}+C_{2}\right)} \end{array}$$ (22)

$$\begin{array}{c} {SSIM}_{OI-DI}=\frac{\left(2\mu_{OI}\mu_{DI}+C_{1})(2\sigma_{OI,DI}+C_{2}\right)}{\left(\mu_{OI}^{2}+\mu_{DI}^{2}+C_{1})(\sigma_{OI}^{2}+\sigma_{DI}^{2}+C_{2}\right)} \end{array}$$ (23)

where μ denotes the mean intensity, $\sigma^{2}$ the variance, $\sigma_{\left(\cdot ,\cdot \right)}$ the covariance, and *C*_1_ and *C*_2_ are constants to avoid instability.

The numerical results of the MSE, PSNR, and SSIM analyses for both grayscale and color images are presented in Table 9. The encrypted images exhibit large MSE values, low PSNR values, and SSIM values close to zero, indicating significant visual distortion and effective concealment of the plaintext information. Conversely, the decrypted images achieve zero MSE, PSNR values approaching infinity, and SSIM values approaching one, demonstrating lossless reconstruction and confirming the correctness and reversibility of the proposed ECPD-MIE scheme.

**Table 9:** MSE, PSNR, and SSIM analysis.

Types of Images	Images	MSE		PSNR		SSIM	
		${MSE}_{OI-EI}$	${MSE}_{OI-DI}$	${PSNR}_{OI-EI}$	${PSNR}_{OI-DI}$	${SSIM}_{OI-EI}$	${SSIM}_{OI-DI}$
Color	4.1.07	8970.1697	0	8.6418	Inf	0.0101	1
	4.1.08	8907.8468	0	8.6608	Inf	0.0100	1
Grayscale	gray21.512	11392.1062	0	7.5648	Inf	0.0090	1
	motion01.512	10645.2478	0	7.8592	Inf	0.0101	1
	motion04.512	10634.4899	0	7.8636	Inf	0.0103	1
	motion07.512	10562.5015	0	7.8931	Inf	0.0104	1
	motion10.512	10613.6110	0	7.8722	Inf	0.0101	1
	ruler.512	21722.6288	0	4.7617	Inf	0.0073	1
Overall Average		11681.0752	0	7.6397	Inf	0.0097	1

### Information entropy analysis

Information entropy is a statistical measure used to quantify the randomness and unpredictability of the pixel intensity distribution in an encrypted image. An increase in the value of entropy represents a higher level of unpredictability, thereby improving the robustness against attacks based on statistical methods and entropy attacks. In an 8-bit image, the maximum possible value of entropy is 8 bits; hence, a high value of entropy closer to the optimal value represents a high level of encryption with minimal information leakage [58].

Global information entropy of an image can be computed using Shannon’s entropy, which can be defined by Eq. 24 as


$$\begin{array}{c} H_{c}=-\sum_{k=0}^{255}P_{c}(k)\;\log_{2}P_{c}(k) \end{array}$$ (24)

where $P_{c}\left(k\right)$ represents the probability of occurrence of the pixel intensity level *k*, and $H_{c}$ denotes the entropy value in bits.

The computed global entropy values of the plaintext and encrypted grayscale and color images are presented in Table 10. For grayscale images, the entropy is computed directly from the image intensity distribution. For color images, the entropy is computed independently for the red, green, and blue channels, and the average of the three channel entropies is reported. It can be observed that the encrypted images achieve entropy values very close to the theoretical maximum value of 8 bits, indicating excellent randomness and negligible statistical information leakage.

**Table 10:** Global Shannon entropy analysis.

Types of Images	Images	Original	Encrypted
Color	4.1.07	5.8346	7.9972
	4.1.08	6.2700	7.9973
Grayscale	gray21.512	4.3923	7.9994
	motion01.512	6.1422	7.9993
	motion04.512	6.2586	7.9993
	motion07.512	6.2611	7.9993
	motion10.512	6.2635	7.9993
	ruler.512	0.5000	7.9993

Table 11 compares the global information entropy achieved by the proposed ECPD-MIE scheme with representative state-of-the-art image encryption algorithms. Since information entropy is influenced by the image size, the corresponding image dimensions reported in each study are also included to facilitate a fair comparison. For the proposed scheme, the average entropy values are 7.9973 for 256×256 images and 7.9993 for 512×512 images, both of which are very close to the theoretical optimum of 8 bits. These results demonstrate that the proposed ECPD-MIE framework produces highly random ciphertexts and provides strong resistance against entropy-based statistical attacks.

**Table 11:** Comparison of global information entropy of encrypted images with different image sizes.

Algorithms	Image Size	Entropy
[15]	512×512	7.9992
[16]	512×512	7.8102
[17]	512×512	7.9993
[18]	512×512	7.9993
[19]	512×512	7.9993
[20]	512×512	7.9994
[21]	512×512	7.9992
[25]	512×512	7.9999 (RGB)
[26]	512×512	7.9998 (RGB)
[27]	512×512	7.9993
[28]	256×256	7.9972
[29]	512×512	7.9993
[30]	256×256	7.9973
[31]	512×512	7.9994
[32]	512×512	7.9997 (RGB)
[33]	256×256	7.9985
[34]	512×512	7.9994
[35]	512×512	7.9991
[36]	512×512	7.9993
[37]	256×256	7.9918
[38]	512×512	7.9997 (RGB)
[39]	512×512	7.9994
[40]	512×512	7.9995
**Proposed**	256×256	7.9973
	512×512	7.9993

Although global entropy effectively measures the overall randomness of an encrypted image, it does not fully characterize the randomness within local image regions. Therefore, local Shannon entropy is employed to evaluate the statistical randomness of local image blocks by partitioning the encrypted image into multiple non-overlapping blocks and computing the average entropy of these blocks [59]. The local entropy can be defined by Eq. 25 as follows:


$$\begin{array}{c} H_{n,T_{p}}(B)=\frac{1}{n}\sum_{i=1}^{n}H(B_{i}) \end{array}$$ (25)

where *n* is the number of non-overlapping blocks, $T_{p}$ denotes the number of pixels in each block, and $B_{i}$ represents the $i^{th}$ image block.

In this work, each encrypted image is divided into *n* = 30 non-overlapping blocks, with each block containing $T_{p}=1936$ pixels. For significance level of 0.05, the acceptable range of local entropies for the (30, 1936) configuration is (7.901901305, 7.903037329). The corresponding acceptable ranges for significance levels of 0.01 and 0.001 are (7.901722822, 7.903215812) and (7.901515698, 7.903422936), respectively.

The local Shannon entropy results presented in Table 12 fall within the acceptable ranges corresponding to all three significance levels (0.05, 0.01, and 0.001). These results demonstrate that the encrypted images exhibit excellent local randomness and uniform statistical behavior throughout the image. Consequently, both the global and local entropy analyses confirm that the proposed ECPD-MIE scheme achieves high randomness at both global and local levels, thereby providing strong resistance against entropy-based statistical attacks.

**Table 12:** Local shannon entropy analysis.

Types of Images	Images	Local Shannon Entropy	Test Results at Significant Levels		
			0.05	0.01	0.001
Color	4.1.07	7.9023	Pass	Pass	Pass
	4.1.08	7.9030	Pass	Pass	Pass
Grayscale	gray21.512	7.9029	Pass	Pass	Pass
	motion01.512	7.9022	Pass	Pass	Pass
	motion04.512	7.9024	Pass	Pass	Pass
	motion07.512	7.9027	Pass	Pass	Pass
	motion10.512	7.9024	Pass	Pass	Pass
	ruler.512	7.9019	Pass	Pass	Pass
Average Local Shannon Entropy		7.9025	Pass	Pass	Pass

### Differential attack analysis

Differential attack analysis is employed to evaluate the sensitivity of an image encryption algorithm to slight variations in the plaintext image. A robust image encryption algorithm should exhibit high sensitivity; this means that a slight change to the plaintext image (for example, by changing a single pixel) should result in a significantly different ciphertext image.

To assess this, two metrics that are commonly used are discussed, namely the Number of Pixel Change Rate (NPCR) and the Unified Average Changing Intensity (UACI), which are determined by introducing a pixel change in the original plaintext image and comparing the resultant encrypted image [60, 61].

The NPCR is a measure of the percentage of pixels that are changed due to a small variation in the plaintext, and it is given by the Eq 26


$$\begin{array}{c} NPCR\left(C_{a},C_{m}\right)=\frac{\sum_{i,j}D\left(i,j\right)}{M\times N}\times 100 \end{array}$$ (26)

where the difference matrix D(i,j) is given by the Eq 27


$$\begin{array}{c} D\left(i,j\right)=\left\{ \begin{array}{cc} 1, & if\;C_{a}\left(i,j\right)\neq C_{m}\left(i,j\right)\\ 0, & otherwise \end{array}\right. \end{array}$$ (27)

Here, $C_{a}$ and $C_{m}$ are the two different ciphertexts produced by a variation in the plaintext, and M×N is the total number of pixels in the image. The NPCR is thus expected to be close to 99.6094% for an ideal 8-bit image encryption scheme, which implies that all the pixels are changed due to a variation in the plaintext [50,61].

The UACI is a measure of the average intensity difference between two different encrypted images, and it is given by the Eq 28


$$\begin{array}{c} UACI\left(C_{a},C_{m}\right)=\frac{1}{M\times N}\sum_{i,j}\frac{\left|C_{a}\left(i,j\right)-C_{m}\left(i,j\right)\right|}{255}\times 100 \end{array}$$ (28)

This is thus expected to be close to 33.4635% for an ideal 8-bit image encryption scheme, which implies that all the intensity values are changed [50,61].

Table 13 shows the NPCR and UACI results for the proposed ECPD-MIE scheme. The observed values closely resemble theoretical values, which confirm the high sensitivity of the algorithm to the changes in the plaintext. This shows that a change in a single pixel can cause a significant change in the ciphertext. To ensure statistical reliability, NPCR and UACI values are calculated based on 100 experiments with randomly altered pixels. In addition to the average values, the statistical dispersion of the obtained results is evaluated through the standard deviation across heterogeneous image sets. Table 13 presents the average NPCR and UACI values for individual test images, whereas Table 14 summarizes the overall statistical characteristics of the obtained results. As shown in Table 14, the standard deviations of NPCR and UACI are only 0.0040% and 0.0023%, respectively. These small variations indicate that the proposed ECPD-MIE scheme exhibits highly consistent differential attack performance across heterogeneous grayscale and color image sets. Furthermore, the obtained NPCR and UACI values remain very close to their theoretical expectations, thereby confirming the robustness of the proposed cryptosystem against differential attacks.

**Table 13:** Differential attack analysis.

Types of Images	Images	Average NPCR (%)	Average UACI (%)
Color	4.1.07	99.6165	33.4724
	4.1.08	99.6186	33.4714
Grayscale	gray21.512	99.6175	33.4722
	motion01.512	99.6107	33.4710
	motion04.512	99.6119	33.4782
	motion07.512	99.6197	33.4716
	motion10.512	99.6195	33.4738
	ruler.512	99.6100	33.4732
Overall Average		99.6155	33.4730
Standard Deviation		0.0040	0.0023

**Table 14:** Statistical summary of NPCR and UACI values across heterogeneous image sets.

Metric	Mean (%)	Standard Deviation (%)
NPCR	99.6155	0.0040
UACI	33.4730	0.0023

Additionally, Table 15 presents a comparative analysis of the proposed method with various state-of-the-art image encryption techniques. As depicted in Table 15, the proposed method achieves competitive NPCR and UACI values compared to various state-of-the-art image encryption techniques. Although some existing methods report slightly higher NPCR or UACI values, the proposed ECPD-MIE scheme provides balanced differential attack performance together with very small statistical variations across heterogeneous image sets. The combination of high average NPCR/UACI values and low standard deviations demonstrates the stability, consistency, and robustness of the proposed cryptosystem against differential attacks.

**Table 15:** Comparison of differential attack analysis.

Algorithms	Average NPCR (%)	Average UACI (%)
[17]	99.6175	33.4950
[18]	99.6165	33.4438
[19]	99.6225	33.4375
[20]	99.6229	33.4809
[21]	99.6066	33.4603
[25]	99.6100	33.4800
[26]	99.6060	33.5126
[27]	99.6142	33.4656
[28]	Pass	Pass
[29]	99.6085	33.4634
[30]	99.6083	33.4211
[31]	99.6143	33.4681
[32]	99.6200	33.6400
[33]	99.6089	33.4658
[34]	99.6190	33.4931
[35]	99.6076	30.6095
[36]	99.6149	33.4585
[37]	99.6086	33.4663
[38]	99.6162	33.4641
[39]	99.6129	33.4577
[40]	99.6020	33.4760
**Proposed**	99.6155	33.4730

### Key sensitivity analysis

Key sensitivity analysis evaluates the impact of slight variations in secret key parameters on the resulting encrypted images. A secure encryption algorithm must exhibit high key sensitivity, such that even an infinitesimal change in any key parameter produces a completely different ciphertext.

In the proposed ECPD-MIE scheme, the secret keys are dynamically generated using a combination of PWLCM-based chaotic parameters and the SHA-256 hash of the plaintext image. Due to the inherent sensitivity of chaotic systems to initial conditions and the strong avalanche effect of the hash function, even a minimal variation in any key parameter leads to a significantly different chaotic sequence. Consequently, the generated ciphertext becomes entirely uncorrelated with that produced using the original key.

To evaluate this property, each key parameter is perturbed independently while keeping all other parameters constant. In this study, a very small perturbation of 10^-15^ is introduced to all chaotic initial conditions and control parameters. The quantitative evaluation is performed for all key parameters, including $\left(v_{1},a_{1}),\;(w_{1},b_{1}),\;(x_{1},c_{1}),\;(y_{1},d_{1}),\;(z_{1},e_{1}\right)$, to comprehensively assess the sensitivity of the proposed encryption scheme.

**Fig 6:**
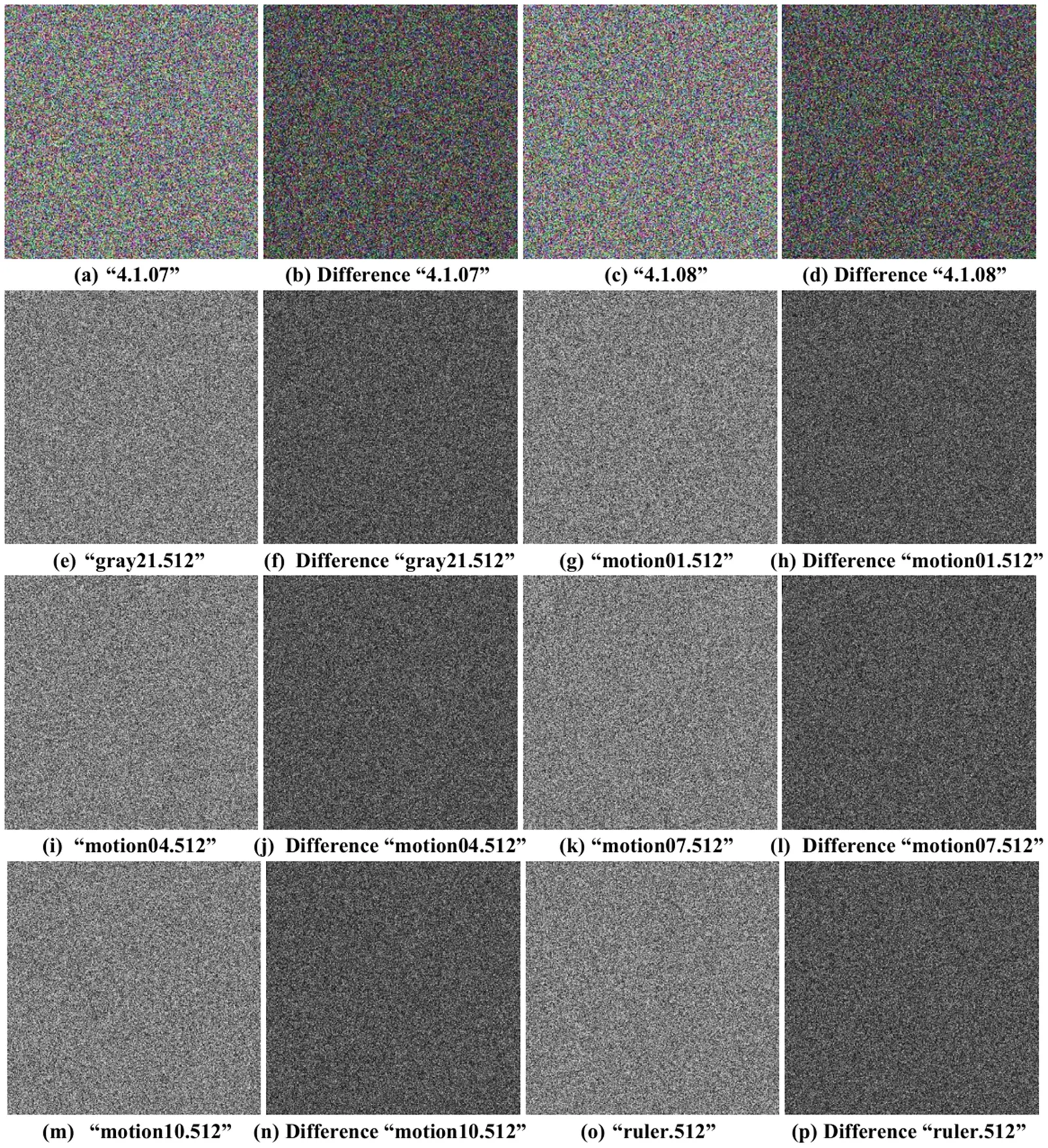
Key sensitivity results in changing key from *z*_1_ to $z_{1}+10^{-15}$.

**Fig 7:**
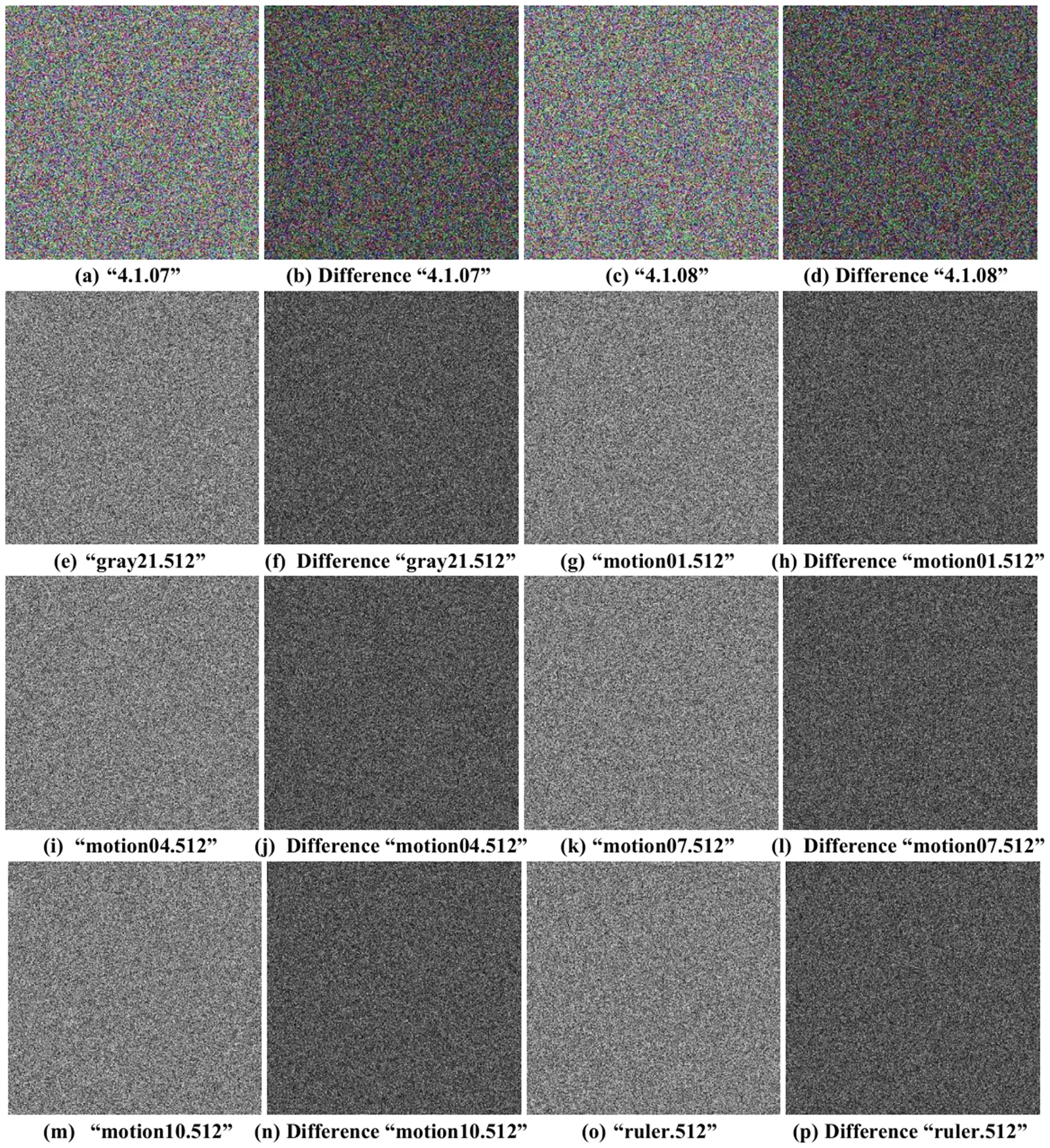
Key sensitivity results in changing key from *e*_1_ to $e_{1}+10^{-15}$.

For instance, only representative key parameters *z*_1_ and *e*_1_ are considered for the purpose of illustration, as all the key parameters show similar characteristics of sensitivity. Figs 6 and 7 show the visual results of the sensitivity analysis of the key parameters for grayscale and color images, respectively. The results show that even a slight change in the key parameters leads to a highly dissimilar encrypted image, which does not show any visual similarity to the encrypted images obtained by using the original keys. The difference images confirm this as the changes are uniformly distributed over the entire image.

For quantitative analysis, the NPCR, UACI, and PSNR measures are obtained between the ciphertexts obtained using the original key and perturbed keys. The results obtained are shown in Table 16. The NPCR and UACI measures are close to their respective ideal values (approximately 99% and 33%, respectively), implying a high degree of pixel-level changes due to key perturbation. Moreover, the PSNR measures are found to be very low, further confirming the high degree of dissimilarity between the obtained ciphertexts using similar keys.

**Table 16:** Key sensitivity analysis.

Types of Images	Images	$v1+10^{-15}$			$a1+10^{-15}$			$w1+10^{-15}$		
		NPCR	UACI	PSNR	NPCR	UACI	PSNR	NPCR	UACI	PSNR
Color	4.1.07	99.5401	33.1156	7.5172	99.7533	33.8283	7.6230	99.2070	33.1508	7.2938
	4.1.08	99.9211	33.5001	7.6951	99.7995	33.2579	7.7639	99.5264	33.7278	7.5656
Grayscale	gray21.512	99.6561	33.3867	7.2769	99.3991	33.9749	7.1696	99.8678	33.3791	7.0153
	motion01.512	99.3169	33.7081	7.9903	99.6638	33.5523	7.0688	99.6510	33.7315	7.4262
	motion04.512	99.9673	33.1823	7.8145	99.3199	33.9732	7.9145	99.9493	33.1563	7.2986
	motion07.512	99.8635	33.2840	7.7231	99.7712	33.3444	7.6719	99.8402	33.1775	7.2666
	motion10.512	99.2417	33.0533	7.8762	99.1014	33.0655	7.8685	99.9432	33.1987	7.2809
	ruler.512	99.0435	33.5121	7.3398	99.8063	33.4346	7.3877	99.6333	33.3017	7.0605
Average		99.5688	33.3428	7.6541	99.5768	33.5539	7.5585	99.7023	33.3529	7.2759
**Types of Images**	**Images**	$b1+10^{-15}$			$x1+10^{-15}$			$c1+10^{-15}$		
		**NPCR**	**UACI**	**PSNR**	**NPCR**	**UACI**	**PSNR**	**NPCR**	**UACI**	**PSNR**
Color	4.1.07	99.5478	33.9597	7.3471	99.5834	33.4637	7.7507	99.5962	33.4093	7.7597
	4.1.08	99.7696	33.5789	7.5969	99.5880	33.4287	7.7513	99.6012	33.4837	7.7458
Grayscale	gray21.512	99.9313	33.1283	7.0533	99.6120	33.3954	7.7642	99.5899	33.4256	7.7591
	motion01.512	99.5747	33.8010	7.4005	99.6231	33.4469	7.7514	99.6254	33.4737	7.7438
	motion04.512	99.9727	33.9670	7.3565	99.5953	33.5053	7.7362	99.5918	33.4751	7.7359
	motion07.512	99.1482	33.1458	7.2673	99.6048	33.4207	7.7570	99.5888	33.3918	7.7620
	motion10.512	99.3321	33.1155	7.3008	99.5815	33.4883	7.7388	99.6029	33.4179	7.7563
	ruler.512	99.5532	33.3248	7.0437	99.6117	33.5194	7.7405	99.6151	33.4485	7.7466
Average		99.6037	33.5026	7.2958	99.6000	33.4585	7.7488	99.6014	33.4407	7.7511
**Types of Images**	**Images**	$y1+10^{-15}$			$d1+10^{-15}$			$z1+10^{-15}$		
		**NPCR**	**UACI**	**PSNR**	**NPCR**	**UACI**	**PSNR**	**NPCR**	**UACI**	**PSNR**
Color	4.1.07	99.5794	33.4731	7.7430	99.5906	33.3763	7.7597	99.5946	33.4473	7.7458
	4.1.08	99.5717	33.4234	7.7529	99.5855	33.4669	7.7514	99.6109	33.5742	7.7222
Grayscale	gray21.512	99.5953	33.4409	7.7544	99.6120	33.5066	7.7427	99.6235	33.5394	7.7338
	motion01.512	99.5785	33.4235	7.7550	99.5937	33.4890	7.7383	99.6067	33.5154	7.7421
	motion04.512	99.5907	33.5015	7.7417	99.5926	33.5502	7.7290	99.6212	33.4987	7.7427
	motion07.512	99.5831	33.4748	7.7444	99.5995	33.4559	7.7510	99.5995	33.3933	7.7600
	motion10.512	99.5995	33.4199	7.7537	99.6109	33.4388	7.7522	99.5983	33.4257	7.7528
	ruler.512	99.6033	33.5609	7.7306	99.5972	33.4740	7.7386	99.6334	33.4530	7.7494
Average		99.5877	33.4647	7.7470	99.5977	33.4697	7.7454	99.6110	33.4809	7.7436
**Types of Images**	**Images**	$e1+10^{-15}$								
		**NPCR**	**UACI**	**PSNR**						
Color	4.1.07	99.5783	33.4750	7.7439						
	4.1.08	99.5682	33.4340	7.7498						
Grayscale	gray21.512	99.5613	33.3986	7.7630						
	motion01.512	99.5907	33.4673	7.7419						
	motion04.512	99.5804	33.4745	7.7419						
	motion07.512	99.6090	33.5320	7.7318						
	motion10.512	99.5983	33.5516	7.7268						
	ruler.512	99.6075	33.4770	7.7413						
Average		99.5867	33.4762	7.7426						

Overall, both the visual and quantitative analyses suggest the presence of key sensitivity for the proposed ECPD-MIE approach. The considerable changes observed in the resulting ciphertexts upon slight modifications to the key may be attributed to the robustness offered by the proposed approach against brute-force attacks and other key-related and differential cryptanalyses.

### Noise attack analysis

In actual communication and storage situations, encrypted images often receive different kinds of noise due to channel interference, transmission errors, and other disturbances. Therefore, besides ensuring security, it is also important for an image encryption algorithm to ensure resilience to noise attacks [62]. Noise attack analysis of an image encryption technique checks for the resilience of the encryption technique in reconstructing acceptable plaintext images from noisy ciphertexts.

To assess the robustness of the proposed ECPD-MIE method, noise is added to the fused encrypted image, and the resulting noisy ciphertext is decrypted using appropriate secret keys. The resulting composite image is then decomposed into individual grayscale and color images for evaluation. In this research, two different noise models are taken into consideration, which are widely used in image processing. They are Gaussian noise and salt & pepper noise.

The quality of the decrypted images is quantitatively evaluated using metrics such as MSE, PSNR, and SSIM in comparison to their corresponding original plaintext images. In this regard, a smaller value of MSE indicates a smaller reconstruction error, and larger values of PSNR and SSIM imply better image quality.

**Fig 8:**
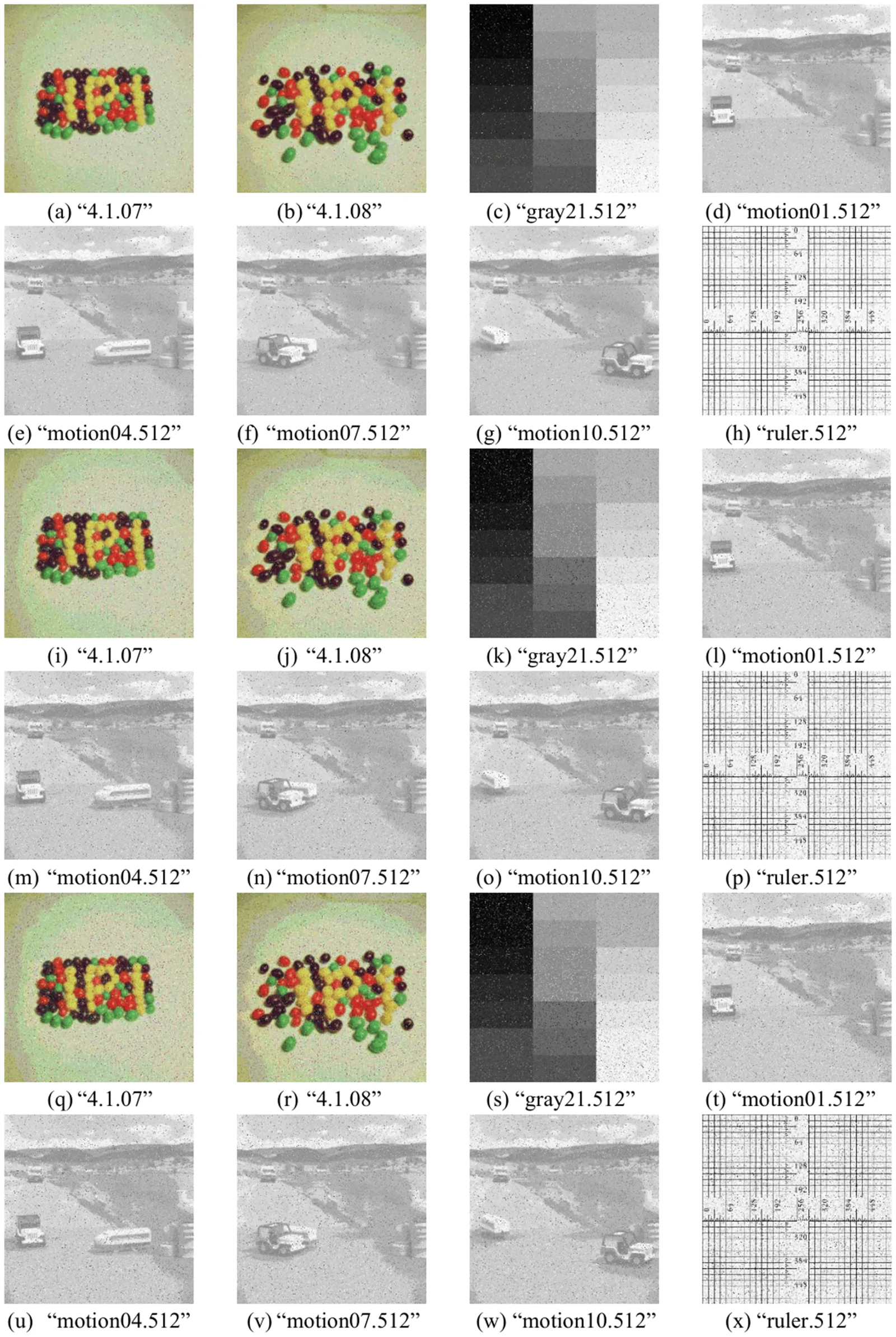
Gaussian noise attack results: (a-h) variance  =  0.0001, (i-p) variance  =  0.0003, (q-x) variance  =  0.0005.

The results of the Gaussian noise perturbation are shown in Fig 8, where Gaussian noise of variances 0.0001, 0.0003, and 0.0005 is added to the encrypted image. The subplots of Fig 8(a-h), Fig 8(i-p), and Fig 8(q-x) represent the images obtained by adding Gaussian noise of variances 0.0001, 0.0003, and 0.0005, respectively. Even when the intensity of the noise is increased, the quality of the images obtained upon decryption is a testimony to the robustness of the proposed scheme.

Quantitative results are presented in Table 17. The proposed method has achieved low MSE values along with high PSNR and SSIM values, thereby indicating that the images obtained after the decryption process are similar to the original images even in the presence of noise.

**Table 17:** Gaussian noise attack analysis.

Types of images	Images	Variance	MSE	PSNR	SSIM
Color	4.1.07	0.0001	239.8830	24.3308	0.7297
	4.1.08		253.2028	24.0961	0.7864
Grayscale	gray21.512		359.7181	22.5712	0.3377
	motion01.512		293.0186	23.4619	0.3876
	motion04.512		288.9027	23.5233	0.4024
	motion07.512		283.2270	23.6095	0.3993
	motion10.512		295.7547	23.4215	0.3936
	ruler.512		980.2689	18.2174	0.7100
Average			374.2470	22.9039	0.5183
Color	4.1.07	0.0003	405.7816	22.0479	0.6271
	4.1.08		416.0869	21.9390	0.7016
Grayscale	gray21.512		632.3457	20.1213	0.2096
	motion01.512		491.2060	21.2182	0.2605
	motion04.512		498.3646	21.1553	0.2706
	motion07.512		491.3263	21.2171	0.2674
	motion10.512		503.0595	21.1146	0.2616
	ruler.512		1597.2345	16.0971	0.6476
Average			629.4256	20.6138	0.4057
Color	4.1.07	0.0005	523.5734	20.9410	0.5720
	4.1.08		529.2900	20.8939	0.6532
Grayscale	gray21.512		817.8849	19.0039	0.1668
	motion01.512		648.8940	20.0091	0.2074
	motion04.512		628.0361	20.1510	0.2216
	motion07.512		636.7521	20.0911	0.2157
	motion10.512		645.9300	20.0289	0.2118
	ruler.512		2023.0527	15.0707	0.6152
Average			806.6766	19.5237	0.3580

**Fig 9:**
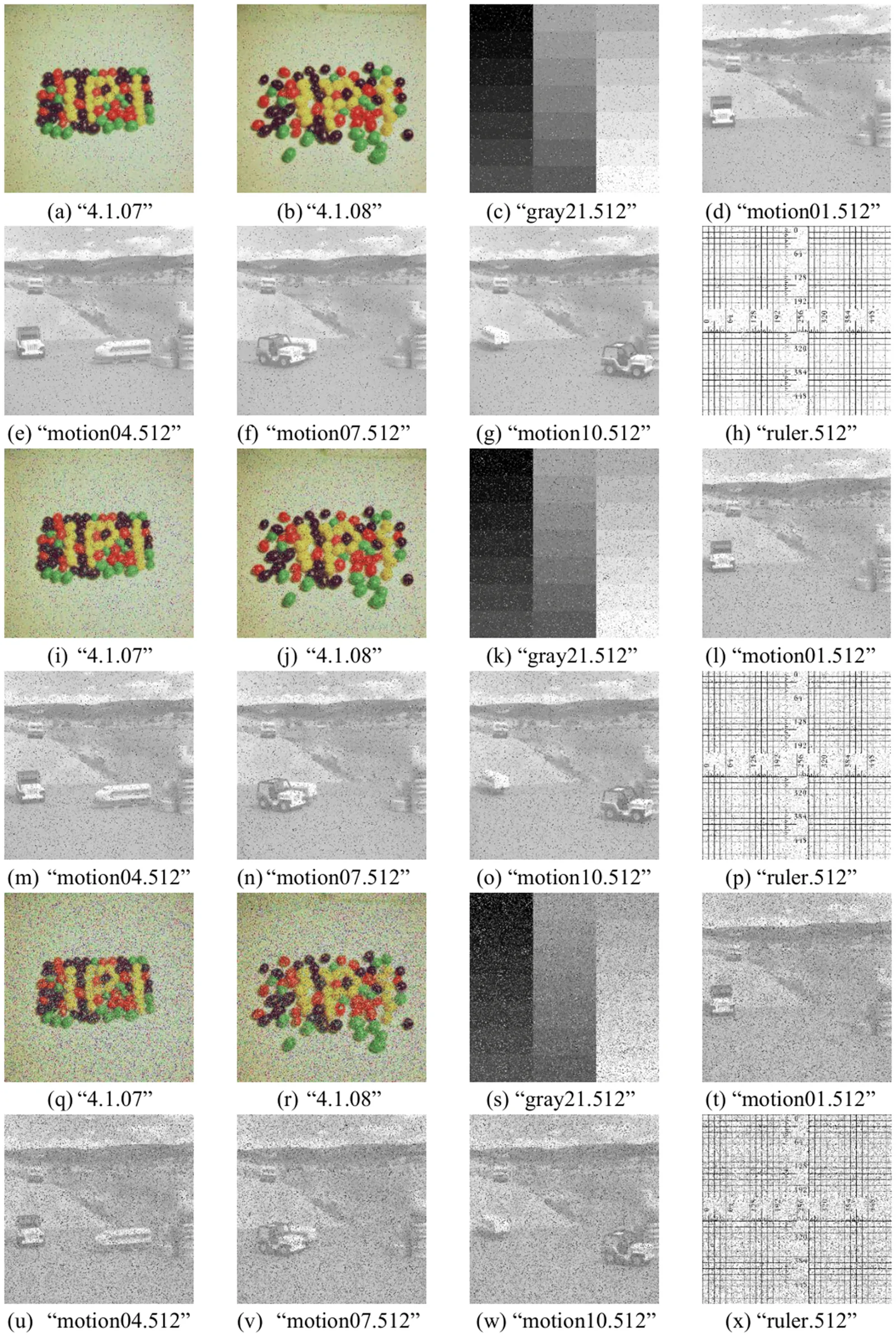
Salt & Pepper noise attack results: (a-h) noise level  =  5%, (i-p) noise level  =  10%, (q-x) noise level  =  25%.

Likewise, the robustness of the proposed scheme in the presence of salt & pepper noise is verified by incorporating noise density levels of 5%, 10%, and 25% in the encrypted image. The corresponding results are depicted in Fig 9, where Fig 9a–9h, 9i–9p, and 9q–9x represent the results for different noise density levels. As depicted, even in the presence of impulse noise, the quality of the decrypted images is satisfactory in terms of structural features.

The quantitative result for the proposed method for the salt & pepper noise attack is shown in Table 18. The result shows that the proposed method has a low MSE value and high PSNR and SSIM values compared to existing methods. This proves that the proposed encryption algorithm can efficiently handle the impact of impulse noise during the transmission process.

**Table 18:** Salt and Pepper noise attack analysis.

Types of Images	Images	Noise Level	MSE	PSNR	SSIM
Color	4.1.07	5%	447.0651	21.6271	0.6321
	4.1.08		436.0881	21.7351	0.7057
Grayscale	gray21.512		577.1607	20.5178	0.3125
	motion01.512		534.3635	20.8524	0.3478
	motion04.512		513.8848	21.0221	0.3705
	motion07.512		533.2609	20.8614	0.3540
	motion10.512		530.0734	20.8874	0.3579
	ruler.512		1066.6654	17.8505	0.7123
Average			579.8202	20.6692	0.4741
Color	4.1.07	10%	904.7536	18.5655	0.4648
	4.1.08		881.0057	18.6810	0.5511
Grayscale	gray21.512		1126.7914	17.6124	0.1414
	motion01.512		1076.9394	17.8089	0.1658
	motion04.512		1063.7772	17.8623	0.1762
	motion07.512		1050.0388	17.9188	0.1750
	motion10.512		1068.6564	17.8424	0.1708
	ruler.512		2149.5517	14.8073	0.6041
Average			1165.1893	17.6373	0.3061
Color	4.1.07	25%	2250.0850	14.6088	0.2619
	4.1.08		2251.5622	14.6060	0.3291
Grayscale	gray21.512		2863.2898	13.5622	0.0440
	motion01.512		2664.4990	13.8746	0.0530
	motion04.512		2691.8799	13.8302	0.0564
	motion07.512		2626.5567	13.9369	0.0568
	motion10.512		2620.1328	13.9476	0.0561
	ruler.512		5428.3047	10.7842	0.4515
Average			2924.5388	13.6438	0.1636

In general, it can be inferred from both visual and quantitative assessments that the proposed ECPD-MIE scheme exhibits excellent resilience under both Gaussian and salt & pepper noise perturbations. The proposed scheme’s ability to produce high-quality decrypted images under noisy circumstances is a testament to the reliability of the proposed framework for image encryption.

### Cropping attack analysis

Cropping attacks, also known as data loss attacks, are commonly used to test the strength of an image encryption system against potential losses of pieces of information during transmission and/or storage. In these types of attacks, a portion of the encrypted image is removed and then decrypted using the relevant decryption keys. A good image encryption system should be able to maintain structural details in the decrypted image even if large pieces of ciphertext are missing.

In this research work, the cropping attacks are carried out on the aggregate encrypted image, which includes both grayscale and color information. In this regard, the excised segments of the ciphertext are cropped from the top-left portion of the aggregate encrypted image. The cropped ciphertext is retained for further investigation. In this regard, the cropping factors 1/16, 1/8, 1/4, and 1/2 are applied to the encrypted image for the simulation of various levels of loss. The cropped ciphertext images are decrypted using the same secret keys as those generated during the encryption process. The cropped encrypted images and their corresponding decrypted outcomes, including the aggregate encrypted image and the grayscale and color components, are retained for further investigation.

**Fig 10:**
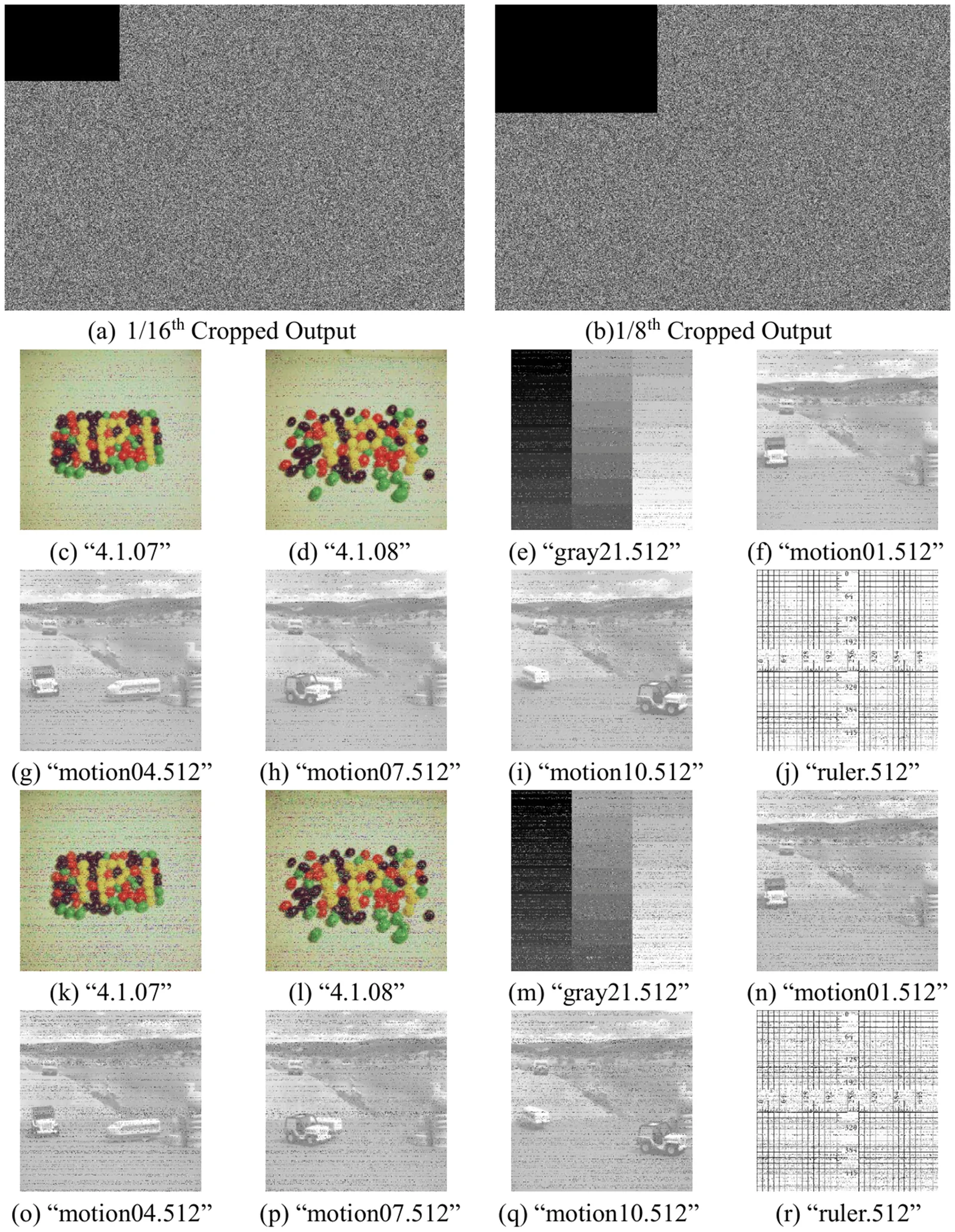
Cropping attack results: (a) 1/16th cropped combined output, (b) 1/8th cropped combined output, (c-j) 1/16th cropped decrypted outputs, (k-r) 1/8th cropped decrypted outputs.

**Fig 11:**
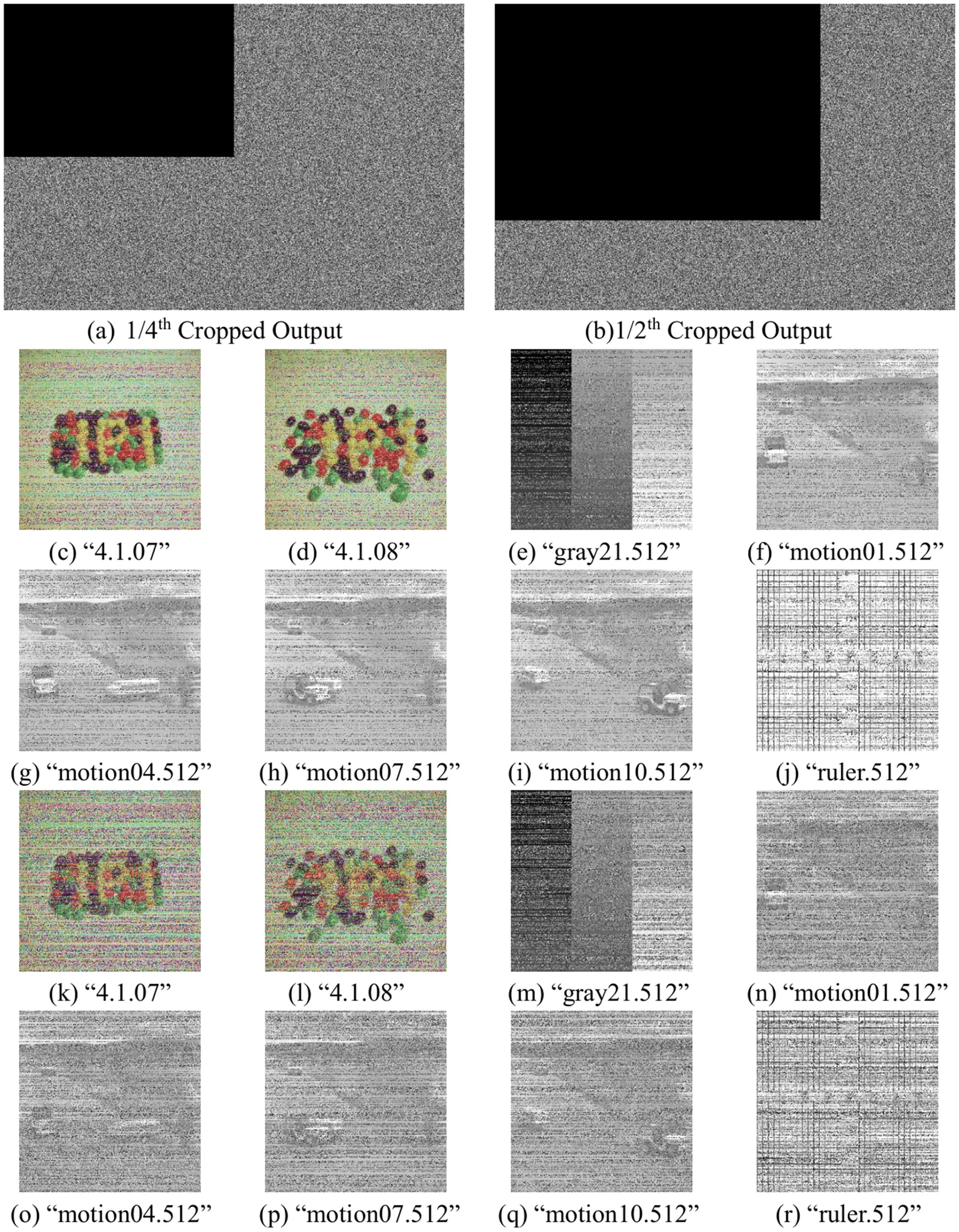
Cropping attack results: (a) 1/4th cropped combined output, (b) 1/2nd cropped combined output, (c-j) 1/4th cropped decrypted outputs, (k-r) 1/2nd cropped decrypted outputs.

The visual results of the cropping attack are shown in Figs 10 and 11. As shown, the decrypted images are the combination of the cropped ciphertext and the decrypted image. Even though the visual quality of the decrypted images is compromised due to the cropping of the ciphertext, the structural and visual information is maintained, even for a cropping factor of 1/2. This is an indicator that the scheme has some redundancy and is robust against the cropping attack.

To perform a quantitative analysis, the PSNR between original images and decrypted images obtained from cropped ciphertexts is calculated. The results for both grayscale and color images are presented in Table 19. As expected, PSNR decreases with an increase in cropping ratios due to increased information loss. However, PSNR remains within an acceptable range for both cases, demonstrating that the quality of decrypted images remains significantly similar to original images.

**Table 19:** Cropping attack analysis.

Types of Images	Images	PSNR (dB)			
		1/16th	1/8th	1/4th	1/2nd
Color	4.1.07	20.6521	17.7252	14.7871	11.6346
	4.1.08	20.5081	17.4367	14.7468	11.6913
Grayscale	gray21.512	19.5370	16.6543	13.3325	10.4969
	motion01.512	20.3345	17.2784	13.9954	10.8395
	motion04.512	19.6768	17.0739	14.0496	10.9096
	motion07.512	20.0809	17.1209	14.0685	11.1127
	motion10.512	19.3899	16.8371	13.7529	10.9654
	ruler.512	16.1130	13.3145	10.5069	7.5722
Overall Average		19.5365	16.6801	13.6549	10.6528

Overall, the experimental outcomes verify the robustness of the proposed ECPD-MIE approach against the cropping attacks. The capability to restore the meaningful image content from the partially disclosed ciphertext validates the reliability and practicability of the proposed multi-image encryption approach.

### Execution time analysis

Execution time analysis represents a significant performance criterion for evaluating the practicality of image encryption algorithms. In addition to ensuring a high level of security, a good encryption technique must also prove its effectiveness in terms of minimal computational overhead and high-speed performance for encryption and decryption operations. In this paper, the performance of the proposed ECPD-MIE technique is evaluated in terms of overall execution time and processing speeds.

In the proposed framework, multiple grayscale and color images are first combined into a single composite image and then processed through a unified encryption structure. Therefore, the reported execution time corresponds to the total time required to encrypt and decrypt all images collectively, rather than processing each image individually. As shown in Table 20, the total encryption time and decryption time for the combined dataset are 15.413728 s and 13.719226 s, respectively.

**Table 20:** Comparison of execution time and processing speed.

Method	System configuration	Image type, No. of images, image size	Encryption time (s)	Decryption time (s)	Encryption speed (MB/s)	Decryption speed (MB/s)
[15]	MATLAB R2012a, Intel Core i5-3570 Processor, 4 GB RAM, 3.4 GHz Frequency	Grayscale, 4, 512×512	9.656 (All Images)	–	–	–
[16]	MATLAB R2012a, Intel Core i5-3570 Processor, 4 GB RAM, 3.4 GHz Frequency	Grayscale, 9, 512×512	0.191 (All Images)	–	–	–
[17]	MATLAB R2012a, Intel Core i5-3570 Processor, 4 GB RAM, 3.4 GHz Frequency	Grayscale, 4, 512×512	0.7103 (All Images)	–	–	–
[18]	MATLAB R2012a, Intel Core i7 Processor, 4 GB RAM, 3.4 GHz Frequency	Color, 8, 512×512	1.623–1.989 (Each Image)	–	–	–
[21]	MATLAB R2016a, Intel Core i5-230M Processor, 4 GB RAM, 2.60 GHz Frequency	Grayscale, 4, 512×512	19.1744 (All Images)	–	–	–
[25]	Intel Xeon W-2133 Processor, 32 GB RAM, 3.60 GHz (Fujitsu Celsius Workstation)	Color, 8, 512×512	1.78 (All Images)	–	–	–
[26]	MATLAB R2016a, Intel M-5Y71 Processor, 8 GB RAM, 1.20 GHz Frequency	Grayscale, 4, 512×512	1.71 (Each Image)	–	–	–
[27]	–	Grayscale, 5, 150×200, 200×200, 256×256, 512×512, 768×512	–	–	2.28	2.37
[29]	–	–	–	–	1.84	1.80
[31]	MATLAB R2016a, Intel Core i7-10750H Processor, 16 GB RAM, 2.60 GHz Frequency	Grayscale, 8, 512×512	2.883 (Each Image)	2.381 (Each Image)	–	–
[34]	MATLAB R2022b, Intel Core i5-12450H Processor, 16 GB RAM, 3.30 GHz Frequency	Color, 7, 512×512	3.7899 (Each Image)	3.8051 (Each Image)	11.0821	11.0378
**Proposed**	MATLAB R2025b, Intel Core i5-1135G7 Processor, 8 GB RAM, 2.40 GHz Frequency	Grayscale, 6, 512×512 Color, 2, 256×256	15.413728 (All Images)	13.719226 (All Images)	0.1216	0.1367

To further evaluate efficiency, encryption and decryption speeds are computed as the ratio of the total input data size to the corresponding processing time, expressed in megabytes per second (MB/s), by Eqs 29 and 30 as follows:


$$\begin{array}{c} Encryption\;Speed=Image\;Size/Encryption\;Time \end{array}$$ (29)

$$\begin{array}{c} Decryption\;Speed=Image\;Size/Decryption\;Time \end{array}$$ (30)

Based on the total input size of 1.8750 MB, the proposed scheme achieves an encryption speed of 0.1216 MB/s and a decryption speed of 0.1367 MB/s. These results indicate that the proposed method maintains consistent processing performance for both encryption and decryption operations.

Table 20 shows a comparison of our proposed algorithm with other existing multi-image encryption algorithms. It can be seen that most of the algorithms give their results for execution time based on per image whereas our algorithm gives execution time based on total time to process multiple images. Though there is a difference in the way these algorithms have been evaluated, our proposed method performs well since it can handle heterogeneous images at once.

While some other existing techniques may result in relatively faster performance, their reliance is mainly on the use of a relatively simple architecture, processing of individual images, or security functions with fewer parameters. Conversely, the novel ECPD-MIE technique involves the use of more than one PWLCM chaotic function, generation of keys using SHA-256, and application of entropy-controlled diffusion all in one-pass permutation-diffusion approach.

In addition, unlike other schemes based on DNA coding, compressive sensing, or multiple transformation rounds, the proposed technique does not use iterative or blocked approaches, thus keeping the computational complexity linear. Processing of both gray and colored images under one roof reduces redundancy and increases the scalability of the proposed method.

As a result, the evaluation of the execution time and speed clearly shows that the newly introduced ECPD-MIE model succeeds in maintaining a proper equilibrium between the computation efficiency and the security robustness. The capability to handle diverse images simultaneously makes it applicable in multimedia secure communications.

### NIST statistical attack analysis

Statistical randomness is a fundamental requirement for a secure cryptographic system, since any statistical regularity present in the ciphertext may be exploited to launch statistical attacks. To evaluate the randomness characteristics of the encrypted images generated by the proposed ECPD-MIE scheme, representative statistical tests from the NIST SP800−22 suite are employed. The NIST SP800−22 statistical test suite, developed by the National Institute of Standards and Technology, provides a collection of statistical tests for evaluating the randomness of binary sequences.

Since the NIST tests operate on binary sequences, each encrypted image is converted into a one-dimensional binary sequence by representing every pixel using its 8-bit binary representation. In the proposed ECPD-MIE framework, six grayscale images of size 512×512 and two color images of size 256×256 are combined to construct a unified composite image of dimensions 2048×2048. Consequently, the binary sequence length used for the NIST statistical analysis is


$$\begin{array}{c} L_{b}=2048\times 2048\times 8=33,554,432\text{ bits}. \end{array}$$

The NIST SP800−22 test suite consists of a comprehensive collection of statistical tests designed to evaluate the randomness characteristics of binary sequences. In the revised analysis, the encrypted bit sequences are evaluated using an expanded set of NIST tests, including Frequency (Monobit), Block Frequency, Runs, Longest Run of Ones, Binary Matrix Rank, Discrete Fourier Transform (FFT), Non-Overlapping Template Matching, Overlapping Template Matching, Universal Statistical, Linear Complexity, Serial, Approximate Entropy, Cumulative Sums (Forward), Cumulative Sums (Reverse), and Random Excursions Variant tests.

Each test produces a p-value, which represents the probability that the tested sequence could be generated by a truly random process. According to the NIST SP800−22 standard, a sequence passes a statistical test if its p-value is greater than or equal to 0.01.

The results obtained are presented in Table 21. It can be observed that all computed p-values are greater than the NIST acceptance threshold of 0.01, indicating that the encrypted bit sequences successfully pass all applied statistical tests. The Frequency and Block Frequency tests confirm a balanced distribution of zeros and ones, while the Runs and Longest Run tests verify the randomness of consecutive bit patterns. The Binary Matrix Rank and FFT tests demonstrate the absence of structural dependencies and periodic patterns in the encrypted sequences.

**Table 21:** NIST SP800−22 statistical test results of encrypted images.

Test Name	p-value	Result
Frequency (Monobit) Test	0.5213	Pass
Block Frequency Test	0.3740	Pass
Runs Test	0.9396	Pass
Longest Run of Ones Test	0.6842	Pass
Binary Matrix Rank Test	0.4517	Pass
FFT (Spectral) Test	0.4689	Pass
Non-Overlapping Template Test	0.7325	Pass
Overlapping Template Test	0.6148	Pass
Universal Statistical Test	0.5523	Pass
Linear Complexity Test	0.4871	Pass
Serial Test	0.2102	Pass
Approximate Entropy Test	0.2672	Pass
Cumulative Sums (Forward) Test	0.7814	Pass
Cumulative Sums (Reverse) Test	0.7449	Pass
Random Excursions Variant Test	0.6358	Pass

Furthermore, the Non-Overlapping Template, Overlapping Template, Universal Statistical, Linear Complexity, Serial, and Approximate Entropy tests confirm the high complexity and unpredictability of the generated ciphertext. The Cumulative Sums and Random Excursions Variant tests further demonstrate the absence of statistically significant deviations from the behavior expected of truly random sequences.

Overall, the successful passing of all applied NIST SP800−22 statistical tests demonstrates that the encrypted outputs generated by the proposed ECPD-MIE scheme exhibit strong statistical randomness, high unpredictability, and no observable statistical bias. These results confirm the suitability of the proposed cryptosystem for resisting statistical attacks and further strengthen the security of the proposed heterogeneous multi-image encryption framework.

### Cryptanalysis

Cryptanalysis is performed for the assessment of security robustness of the proposed ECPD-MIE scheme. A robust image encryption scheme needs to effectively counter statistical, differential, brute-force, and data loss attacks, besides being highly sensitive to the key and maintaining randomness. In this work, a series of cryptanalysis tests are performed on the proposed scheme, which include statistical analysis, assessment of the differential attack, key sensitivity, evaluation of the scheme under noisy and cropped conditions, randomness testing through statistical tests based on the guidelines of the National Institute of Standards and Technology, and assessment of the scheme for classical models of a cryptographic attack. Mathematically, the encryption process is defined as follows:


$$\begin{array}{c} C\;=\;E(P,\;K) \end{array}$$

where *P* is the plaintext image, *C* is the ciphertext image, and *K* is the secret key used for encryption. A robust encryption scheme needs to ensure that a small variation in the plaintext or the key results in a considerably different ciphertext. This is defined as follows:


$$\begin{array}{c} E(P,K)\neq E(P+\Delta P,K) \end{array}$$

$$\begin{array}{c} E(P,K)\neq E(P,K+\Delta K) \end{array}$$

These properties ensure strong sensitivity to both plaintext and key variations, which are validated through the analyses presented in this section.

#### Statistical security analysis.

The statistical properties of the encrypted images are checked by histogram analysis, histogram variance, the chi-square method, pixel correlation, and information entropy. The results show that the encrypted images have a nearly uniform histogram, low histogram variance, and a chi-square value within the statistically significant range. Moreover, the correlation coefficient between two adjacent pixels for the horizontal, vertical, and diagonal directions is close to zero, indicating the elimination of inherent redundancy present in the original image. The global and local entropy values are close to the maximum theoretical value, confirming the high randomness and low information leakage. In summary, the proposed method has been shown to be secure against statistical attacks.

#### Differential attack resistance.

The strength of the proposed scheme against differential attacks is also evaluated based on NPCR and UACI criteria. NPCR values converge towards the optimal value of nearly 99%, whereas the evaluated UACI values approximate the optimal value close to 33%. This implies that any variation in a pixel of the plaintext image results in a substantial change in the corresponding ciphertext, thereby meeting the criterion:


$$\begin{array}{c} E(P,K)\neq E(P+\Delta P,K) \end{array}$$

This demonstrates the strong diffusion characteristics of the scheme.

#### Key space and key sensitivity analysis.

The key space for the proposed scheme is sufficiently large due to the incorporation of multiple chaotic parameters, entropy-based parameters, and hash-based keys, thereby making it more resilient to brute-force attacks. Moreover, key sensitivity analysis is performed by introducing a small perturbation (10^-15^) in each key parameter. The resulting ciphertexts show significant deviations compared to the ciphertexts obtained using the original keys. Quantitative analysis using NPCR, UACI, and PSNR measures also prove that a small change in any key parameter results in a ciphertext that is completely uncorrelated with the original one, satisfying the condition:


$$\begin{array}{c} E(P,K)\neq E(P,K+\Delta K) \end{array}$$

This shows that the proposed scheme is highly sensitive to the key parameters.

#### Noise attack analysis.

The robustness of the proposed encryption method under noisy environments is verified by injecting Gaussian noise and salt & pepper noise into the encrypted images. The decrypted images are obtained by using the correct keys for decryption. The quality of the decrypted images is verified by using the MSE, PSNR, and SSIM. The decrypted images show good quality and structural information even in noisy environments. The high PSNR and SSIM values, along with the low MSE value, verify the robustness of the proposed method under noisy environments.

#### Cropping attack analysis.

Cropping attacks are carried out by removing a part of the composite encrypted image in different proportions. The decrypted images are obtained by decrypting the cropped ciphertext. The decrypted images are analyzed qualitatively and quantitatively. Although the decrypted images suffer from a loss of information, the proposed scheme shows robustness by providing a high PSNR value. This shows that the proposed scheme can provide a high level of security even under a cropping attack.

#### Randomness evaluation using NIST statistical tests.

To further verify the randomness of the encrypted data, a set of statistical tests based on NIST SP800−22 are employed for the binary data obtained from the encrypted images. The tests for frequency, block frequency, runs, longest run, rank, spectral (FFT), approximate entropy, serial, and linear complexity analyses are performed. The values obtained for the tests are well above the significance level of 0.01; therefore, the randomness of the encrypted data is verified. This further affirms that the proposed approach is efficient in removing statistical patterns and improving immunity to sophisticated statistical attacks.

#### Resistance to known-plaintext, chosen-plaintext, and chosen-ciphertext attacks.

In the context of real-life applications of cryptography, the attackers may try to use the existing pairs of plaintext and ciphertext, as well as modify the input to infer the confidential information. In the proposed method, the encryption keys are generated dynamically from the plaintext image by a hash-based method coupled with chaotic parameters. This method guarantees the key stream to have a strong dependency on the input image, making it impossible for the key information to be reused for different images.

In the case of known plaintext attacks, the strong properties of the diffusion and permutation steps hinder the establishment of any relationship between the plaintext and ciphertext pairs, even if the number of pairs is large. In the case of the chosen plaintext attack, the most common relationship between the plaintext and key is dynamic, meaning that any plaintext of discerning choice will have ciphertext that is significantly different, i.e., $E(P_{1},K)\neq E(P_{2},K)$ for any two plaintexts *P*_1_ and *P*_2_.

Likewise, resistance against chosen ciphertext attacks (CCA) is achieved since the decryption process is exclusively dependent on the key parameters and chaotic sequences. Any slight variation in the ciphertext or key parameters will cause a significant distortion in the decrypted image. This implies that attackers will not be able to obtain any useful information through manipulating the ciphertext.

The extensive cryptanalysis has shown that the proposed scheme of ECPD-MIE provides strong security against a wide range of attacks, including statistical, differential, brute-force, noise-related, data loss, and conventional cryptographic attacks. It is evident that the scheme provides substantial randomness, strong diffusion, and key sensitivity, which make the scheme appropriate for secure multi-image encryption in practical scenarios.

### Computational complexity analysis

The complexity of the proposed ECPD-MIE algorithm depends on three main processes: the generation of the chaotic sequence, the permutation-diffusion process, and the permutation step. If we assume that the size of the concatenated image, consisting of grayscale and color images, is M×N, then we have L=M×N representing the total pixel count. In the case of color images, the *RGB* components are included in the proposed structure; however, the complexity is determined by the total pixel count *L*.

#### Chaotic sequence generation.

The proposed scheme employs five PWLCM systems for generating the required pseudo-random sequences for the permutation and diffusion stages. PWLCM-1 and PWLCM-2 are employed for the row and column permutations, where the number of iterations required is *M* and *N*, respectively. Similarly, PWLCM-3, PWLCM-4, and PWLCM-5 are employed for the diffusion stage, where the required iterations for each system are *L*. Thus, the total iterations required for the chaotic sequence generation can be given by the equation:


$$\begin{array}{c} f(M,\;N,\;L)\;=\;3L\;+\;M\;+\;N \end{array}$$

As L≫M,N, the equation can be approximated as:


$$\begin{array}{c} f(M,\;N,\;L)\;\approx \;3L\;\approx \;L \end{array}$$

As the number of iterations for each PWLCM system is constant, the computational complexity for the chaotic sequence generation can be given by:


$$\begin{array}{c} O\left(L\right)=O(M\;\times \;N) \end{array}$$

#### Permutation complexity.

The permutation stage includes chaotic circular permutations for the concatenated image along both the row and column axes. In the row permutation stage, each of the *M* rows are subject to a circular shift over the *N* columns, while in the column permutation stage, each of the *N* columns are subject to a circular shift over the *M* rows. Since each pixel is accessed and shifted only once for each permutation stage, and the computational efficiency of circular shift functions, the computational complexity for the permutation stage can be given by:


$$\begin{array}{c} O\left(L\right)=O(M\;\times \;N) \end{array}$$

#### Diffusion complexity.

The diffusion stage implements entropy-controlled masking and cross-bit-plane diffusion using various chaotic sequences. The XOR operation is performed on the pixel by a predetermined number of chaotic matrices and the entropy weight factors. The number of operations on the pixel is constant, and the complexity of the diffusion is given by the formula:


$$\begin{array}{c} O(L)\;=\;O(M\;\times \;N) \end{array}$$

#### Overall encryption and decryption complexity.

Thus, by combining all the stages, the overall computational complexity for the entire encryption procedure can be stated as:


$$\begin{array}{c} O\left(L\right)=O(M\;\times \;N) \end{array}$$

It should be noted that the proposed ECPD-MIE scheme processes a unified composite image rather than encrypting individual images separately. Therefore, the computational complexity depends on the total number of pixels in the composite image rather than on the number of constituent images. If multiple heterogeneous images are combined to form a composite image of dimensions M×N, the permutation and diffusion stages are executed only once on the resulting composite image. Consequently, the computational complexity remains O(M×N) irrespective of the number of grayscale and color images included in the composite image.

The decryption procedure involves the use of the inverse diffusion and the inverse permutation. This has the same computational complexity as the encryption procedure. Thus, the computational complexity for the decryption procedure can be stated as:


$$\begin{array}{c} O\left(L\right)=O(M\;\times \;N) \end{array}$$

Similarly, during decryption, the chaotic diffusion sequences generated by PWLCM-3, PWLCM-4, and PWLCM-5 are regenerated only once for the entire composite image. Therefore, even when encrypting or decrypting a large number of heterogeneous images, the computational cost scales linearly with the total pixel count rather than with the number of images. For example, a set of 100 heterogeneous images having a combined size of 2048×2048 pixels requires processing of only 4,194,304 pixels, thereby preserving the linear complexity of the proposed scheme.

The proposed ECPD-MIE scheme follows a single-pass permutation-diffusion architecture, which eliminates the need for iterative rounds, block-wise transformations, or computationally expensive operations such as sorting, matrix decomposition, or transform-domain processing.

From the comparative analysis presented in Table 22, it can be observed that:

**Table 22:** Comparative computational complexity analysis of multi-image encryption schemes.

Algorithms	Core technique	Complexity	Remarks
[15]	Bit-plane decomposition + chaos	*O*(*L*)	Bit-level operations
[16]	Mixed image element + chaos	*O*(*L*)	Pixel-wise permutation
[17]	Permutation-based MIE	O(LlogL)	Sorting-based permutation
[18]	Multi-level permutation–diffusion	*O*(*L*)	Efficient linear
[19]	DNA encoding + chaos	O(LlogL)	DNA + sorting overhead
[20]	Dual-layer chaotic MIE	*O*(*L*)	Multi-layer PD
[21]	Hash + bit-plane + DNA	*O*(*L*)	Bit-level overhead
[25]	3D scrambling + hyperchaos	*O*(*L*)	Sequential
[26]	3D scrambling + DNA	*O*(*L*)	Bit-plane operations
[27]	Single-channel encryption	*O*(*L*)	Reduced data size
[28]	Perturbed chaotic map	*O*(*L*)	Higher constant cost
[29]	Cross-plane encryption	*O*(*L*)	Efficient structure
[30]	Quaternion transform	O(LlogL)	Transform overhead
[31]	ROI-based encryption	*O*(*L*)	Selective processing
[32]	ESC chaos + DNA diffusion	*O*(*L*)	DNA constant overhead
[33]	DWT + bit-level encryption	O(LlogL)	DWT dominates
[34]	Parallel DNA cross-plane	*O*(*L*)	Parallel optimized
[35]	Hyperchaos + SVD + RC5	O(LlogL)	SVD dominates
[36]	Spatiotemporal chaos	*O*(*L*)	Iterative linear
[37]	4D chaos + embedding	*O*(*L*)	Block processing
[38]	Parallel diffusion MIE	*O*(*L*)	High-speed design
[39]	BP neural network + chaos	O(LlogL)	Neural overhead
[40]	DNA + FPGA implementation	*O*(*L*)	Hardware acceleration
**Proposed**	Entropy-controlled permutation-diffusion	*O*(*L*)	Optimal + low overhead

- Most modern chaos-based multi-image encryption schemes achieve linear complexity *O*(*L*), primarily due to pixel-wise permutation-diffusion structures.
- However, schemes incorporating transform-domain operations (e.g., DWT, quaternion transforms), matrix decompositions (e.g., SVD), compressive sensing, or neural network-based processing exhibit higher computational complexity of O(LlogL) due to additional mathematical operations.
- DNA-based schemes, although theoretically linear, introduce higher computational overhead due to encoding and decoding operations at the bit level.

In contrast, the proposed ECPD-MIE scheme avoids such computationally intensive operations and processes both grayscale and color images within a unified framework using a single-pass structure.

Furthermore, the use of a unified composite-image framework enables efficient scalability for large heterogeneous image sets, since the encryption and decryption procedures depend on the total number of pixels rather than the number of individual images.

### Comparison analysis

To further verify the effectiveness of the proposed ECPD-MIE scheme, a comprehensive comparison with several state-of-the-art image encryption schemes is conducted. The results of this comparison are presented in Table 20. It summarizes the total time required for encryption and decryption, and the speed of both processes under similar system conditions.

Based on the outcomes, it is obvious that the proposed scheme achieves competitive execution times along with high processing speeds. The increased efficiency of the proposed method can be attributed to the single-pass permutation-diffusion configuration and the use of a unified image processing approach, which avoids the need for repeated passes, block-based processing, and the differentiation between grayscale and color images. This method greatly minimizes the computational cost compared to a wide variety of existing methods.

On the contrary, a variety of conventional encryption techniques are based on multi-round permutation-diffusion structures, DNA encoding operations, or compressive sensing methods, which increase the complexity of computation and delay the computation process. While some techniques may provide faster computation in specific scenarios, they often compromise scalability or ease of implementation. The proposed ECPD-MIE scheme provides a balanced trade-off between efficiency and security.

Besides, as opposed to many existing techniques which deal with grayscale and color images separately, the proposed method efficiently handles several heterogeneous images within a unified framework, thus improving the processing efficiency and reducing redundancy. This unified processing scheme improves the temporal efficiency and simplicity of implementation, making it suitable for real-time processing.

Apart from that, the scheme also demonstrates a strong security feature, as shown by statistical analysis, resistance to differential attacks (NPCR and UACI), key sensitivity, robustness against noise and cropping attacks, and randomness testing based on the NIST model. It is possible that some schemes may perform better than the proposed scheme in a particular criterion, but they may not perform well in all criteria.

From the above comparative analysis, it can be concluded that the proposed ECPD-MIE scheme offers a good compromise between computational efficiency and cryptographic strength. The linear time complexity, fast computing capability, and robust security features of the proposed scheme make it a viable option for secure multi-image encryption in communication systems.

## Conclusion

This research proposes a new method of multi-image encryption, called ECPD-MIE (Entropy-Controlled Chaotic Permutation-Diffusion based Multi-Image Encryption), for secure and efficient encryption of both grayscale and color images. The new method is based on chaotic systems, entropy-controlled masking, and hash-based key generation, which ensure both security and efficiency. Unlike traditional image encryption schemes, which often deal with images individually or require multi-round encryption, the new method is based on a combination of images and a single-round permutation-diffusion architecture, making it possible to encrypt multiple heterogeneous images simultaneously. This not only makes the encryption process much simpler but also helps in reducing computational complexity, making it more suitable for real-time applications.

A comprehensive set of experimental and analytical evaluations has been conducted to ascertain and validate the effectiveness of the proposed method. It has been observed from the results of various statistical tests, namely histogram test, entropy test, chi-square test, and pixel correlation test, that the resulting encrypted images possess high randomness with very low statistical leakage. Moreover, the results of the differential test demonstrate high sensitivity of the method to changes in plaintext images. The NPCR and UACI results are very close to their ideal limits. The key sensitivity test results verify that even with minor variations in key parameters, significantly different ciphertexts are produced. It has also been demonstrated that the proposed method is robust against noise and cropping attacks. Moreover, various statistical test results based on NIST SP 800−22 guidelines verify high random characteristics of the method. It has also been demonstrated that the proposed method is immune to known-plaintext attacks, chosen-plaintext attacks, and chosen-ciphertext attacks. The time complexity of the proposed method is linear, i.e., O(M×N), and its execution time is competitive with high processing speed. Thus, considering various aspects of ECPD-MIE, it may be concluded that the proposed method is a promising solution for secure multi-image encryption in modern multimedia communication systems.

Possible future work may be focused on extending the proposed framework for hardware acceleration using FPGAs/GPUs for the execution of real-time applications, video encrypting capabilities, and the integration of other technologies such as the Internet of Things (IoT) and 6G networks. Moreover, the integration of AI-based optimization and post-quantum cryptography can be further included for greater adaptability and security.
